# Spatiotemporal signaling underlies progressive vascular rarefaction in myocardial infarction

**DOI:** 10.1038/s41467-023-44227-6

**Published:** 2023-12-21

**Authors:** Lin Wei Tung, Elena Groppa, Hesham Soliman, Bruce Lin, Chihkai Chang, Chun Wai Cheung, Morten Ritso, David Guo, Lucas Rempel, Sarthak Sinha, Christine Eisner, Julyanne Brassard, Kelly McNagny, Jeff Biernaskie, Fabio Rossi

**Affiliations:** 1 School of Biomedical Engineering & Department of Medical Genetics, University of British Columbia, 2222 Health Sciences Mall, Vancouver, BC V6T 1Z3 Canada; 2 Borea Therapeutics, Scuola Internazionale Superiore di Studi Avanzati, Via Bonomea, 265, 34136 Trieste, Italy; 3 Aspect Biosystems, 1781 W 75th Ave, Vancouver, BC V6P 6P2 Canada; 4 Faculty of Pharmaceutical Sciences, Minia University, Minia, Egypt; 5 Faculty of Veterinary Medicine, University of Calgary, Calgary, AB Canada; 6 Department of Surgery, Cumming School of Medicine, University of Calgary, Calgary, AB Canada

**Keywords:** Cardiac regeneration, Myocardial infarction, Cellular signalling networks

## Abstract

Therapeutic angiogenesis represents a promising avenue to revascularize the ischemic heart. Its limited success is partly due to our poor understanding of the cardiac stroma, specifically mural cells, and their response to ischemic injury. Here, we combine single-cell and positional transcriptomics to assess the behavior of mural cells within the healing heart. In response to myocardial infarction, mural cells adopt an altered state closely associated with the infarct and retain a distinct lineage from fibroblasts. This response is concurrent with vascular rarefaction and reduced vascular coverage by mural cells. Positional transcriptomics reveals that the infarcted heart is governed by regional-dependent and temporally regulated programs. While the remote zone acts as an important source of pro-angiogenic signals, the infarct zone is accentuated by chronic activation of anti-angiogenic, pro-fibrotic, and inflammatory cues. Together, our work unveils the spatiotemporal programs underlying cardiac repair and establishes an association between vascular deterioration and mural cell dysfunction.

## Abstract (web summary)

Enhancing vascularization to improve cardiac disease outcomes is a therapeutic approach with limited success. Here, the authors show that cardiac repair is governed by spatiotemporally regulated programs and underline the signaling mechanisms driving vascular deterioration.

## Introduction

Ischemic heart disease (IHD) is the leading cause of mortality globally^1^. It is characterized by reduced blood perfusion to the cardiac tissue, leading to irreversible myocardial cell death and the development of non-functional scars^2^. With the advancement of human pluripotent stem cell technologies, strategies aimed at replacing cardiomyocytes by transplanting cells directly into the infarct have been explored. However, these approaches are limited by the ability of transplanted cells to survive, which is likely compromised in hypovascularized regions such as the post-MI scar^3^. Indeed, to slow disease progression and improve blood perfusion to the ischemic myocardium, “therapeutic angiogenesis” as a strategy that promotes vessel growth through neovascularization by targeting the vasculature and cardiac stroma has been proposed^4^. One main approach revolves around the administration and targeting of the pro-angiogenic molecule, vascular endothelial growth factor (VEGF)^5^. While VEGF is a potent inducer of angiogenesis, the success of VEGF-based gene therapies is limited as adequate angiogenic response requires tight control of VEGF level and its duration of effect^6,7^. Low or transient expression of VEGF has minimal angiogenic effects, whereas high or prolonged expression induces dysfunctional vessels with structural abnormalities and may result in the development of angioma. Stimulating endothelial tube formation without adequate perivascular cell recruitment, namely mural cells, is also insufficient for building mature, stable vessels^8,9^. More recently, cell-based therapy using mesenchymal stromal cells (MSCs) has emerged as a potential approach to limiting the infarct size through the pro-angiogenic activities of MSCs^10^. However, the results of clinical trials exploring this approach are rarely in agreement. We believe part of this discrepancy stems from the underappreciated phenotypic and functional heterogeneity of stromal cells, which can adopt different functional states over time in response to local stimuli. Thus, elucidating the behavior of these cells in situ and characterizing the signaling programs underlying their environment may better guide future therapeutic attempts.

We and others have identified two major cardiac stromal populations at single-cell resolution, fibroblasts and mural cells, a class of perivascular cells consisting of vascular smooth muscle cells (VSMC) and pericytes^11^. Upon the initial ischemic event during myocardial infarction (MI), fibroblasts drive the formation of a robust scar that maintains cardiac integrity and function. However, prolonged ischemia post infarction (PI) leads to persistent activation of fibroblasts, resulting in chronic myocardial fibrosis. On the other hand, the behavior of mural cells in ischemia is much less characterized. While mural cells are implicated as critical drivers of neovascularization PI^12^, their fate and function within the scar tissue remain elusive. It is also unclear whether they function to potentiate or reduce fibrosis. This is likely due to the use of promiscuous lineage-tracing models and a historical lack of consistent distinction between the main stromal cell types, which until recently precluded our understanding of their roles in tissue repair and lineage relationships. For example, it is unclear if fibroblasts, specifically fibro-adipogenic progenitors (FAPs), given their progenitor-like and multipotent properties^13,14^, can give rise to mural cells after injury. More importantly, our limited knowledge of spatially resolved response and signaling networks with respect to the infarct adds another layer of complexity to dissecting scar formation and its underlying organization.

In the present study, we demonstrated that cardiac mural cells are anatomically distinct from fibroblasts and exhibit unique transcriptional programs at homeostasis. Following MI, pericytes undergo activation and adopt a pathological transcriptional profile characterized by increased matrix remodeling and dysregulated vascular programs. Furthermore, fate mapping of the stromal subsets PI showed that they do not share a lineage relationship. In the infarct zone, ischemia is reflected by vascular rarefaction and dysfunction, which coincides with the loss of vascular coverage by mural cells and their activation. By creating a spatiotemporal transcriptomic atlas, we mapped the signaling dynamics of tissue repair programs and identified potential mechanisms perpetuating ischemia. Overall, our study provides a clear picture of processes underlying the infarcted heart and highlights a relationship between vascularization and mural cell function.

## Results

### Heterogeneity of stromal cells in the heart

**Fig. 1:**
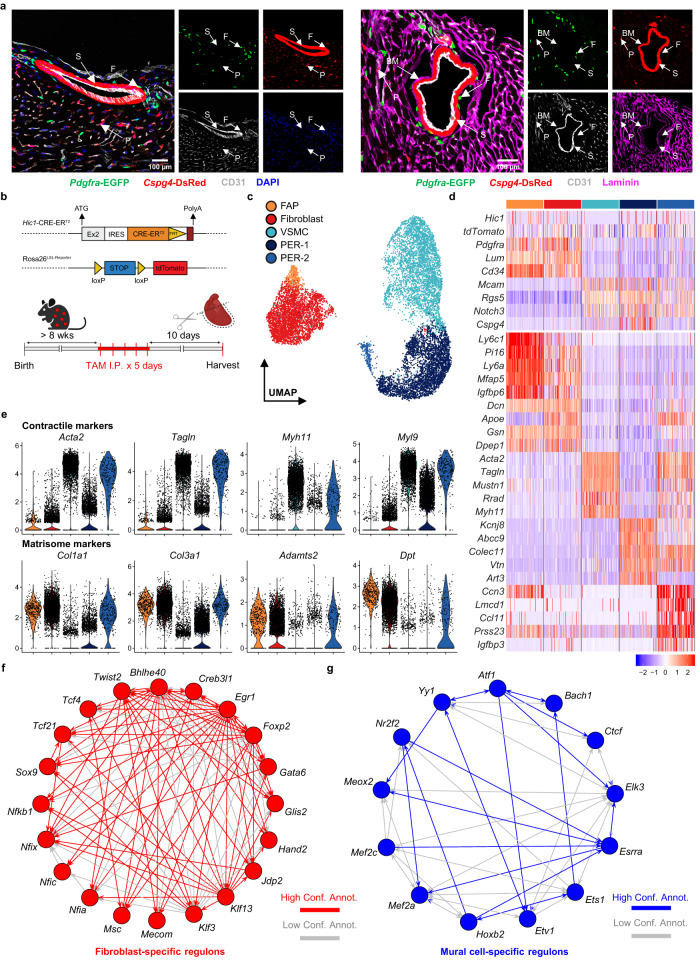
Cardiac fibroblasts and mural cells are distinct stromal entities. **a** Confocal images of fibroblast (EGFP, green) and mural cell (DsRed, red) localization with respect to the vascular endothelium (CD31, gray; left) and basement membrane (Laminin, magenta; right) in *Pdgfra*-EGFP/*Cspg4*-DsRed hearts. Arrows indicate VSMC (S), fibroblast (F), pericyte (P), and basement membrane (BM). DAPI (blue) was used as nuclear counterstain. Scale bar = 100 µm. Representative image of three biological replicates. **b** Graphical illustration of the *Hic1*-CT2/tdTomato reporter construct and experimental scheme. LIN^−^tdTomato^+^ fraction with enrichment for CD146^+^ cells was isolated from heart ventricles of tamoxifen (TAM) treated mice for library preparation. LIN = CD45 and CD31. **c** UMAP projection of 11,489 tdTomato^+^ stromal cells colored by subset annotations. FAP fibro-adipogenic progenitors, VSMC vascular smooth muscle cells, PER pericytes. **d** Heatmap depicting the relative expression levels of selected genes (top) and top five upregulated DEGs per subset (bottom). **e** Violin plots depicting normalized expression of contractile (top) and matrisome markers (bottom) in each annotated subset. **f**, **g** Web diagrams of GRNs preferentially active in fibroblasts (**f**) and mural cells (**g**) inferred from SCENIC. Regulatory links among TFs are distinguished as high or low confidence based on annotation source (database, orthology, and motif similarity). Source data are provided as a Source Data file.

Distinction of mural cells from adventitial fibroblasts has been typically overlooked due to their anatomical proximity around blood vessels and overlapping gene signatures^15,16^. To elucidate their distinction in vivo, we generated a double reporter mouse line, *Pdgfra*-EGFP/*Cspg4*-DsRed, that labels fibroblasts with nuclear EGFP under their canonical marker, platelet-derived growth factor receptor α (*Pdgfra*)^17^, and mural cells with cytoplasmic DsRed under the chondroitin sulfate proteoglycan-4 (*Cspg4*) promoter^18^. Taking advantage of this system, we showed that EGFP and DsRed-expressing cells are clearly distinct in the heart at homeostasis (Fig. 1a, left). In fact, perivascular DsRed^+^ cells are embedded under the vascular basement membrane (BM), whereas EGFP^+^ cells are not (Fig. 1a, right). This is evident in large vessels where DsRed^+^ cells associate closely with endothelial cells, while EGFP^+^ cells are found mostly in the adventitia. The mutual exclusivity of labeled cells and their difference in localization with respect to blood vessels support the notion that mural cells are a distinct cell type from fibroblasts and likely exhibit a different functional role in the heart.

To explore the heterogeneity of cardiac stromal cells, we adopted the established mouse strain, *Hic1*-CT2/tdTomato, that allows for the labeling of multiple stromal subsets in various organ systems^13,19,20^. Following tamoxifen (TAM) administration, *Hic1*-expressing cells and their progeny are labeled with tdTomato (Fig. 1b). Ventricles of the heart were collected at 10 days post-TAM treatment and enzymatically digested. Using fluorescence-activated cell sorting (FACS), tdTomato^+^ cells were isolated for single-cell RNA sequencing (scRNAseq). To ensure high purity and adequate representation of all stromal subsets, in addition to gating out immune cells and endothelial cells, mural cells were enriched based on a pan-mural cell marker, CD146 (*Mcam*)^21^, prior to library preparation. To minimize cross-sample multiplets, we applied cell hashing with oligo-tagged antibodies specific to each mouse sample.

Louvain clustering of 11,489 cells generated 12 clusters (Supplementary Fig. 1a). We noted that cells derived from each mouse were similarly distributed throughout the clusters, suggesting that hashing did not introduce significant sampling bias (Supplementary Fig. 1b). Consistent with what was observed in previous publications^13,19^, *Hic1* transcript expression was low but virtually all cells in the dataset carried *tdTomato* transcripts (97.2%, Supplementary Fig. 1c), validating the purity of sorted cells. To identify distinct cell populations, hierarchical clustering of 12 clusters showed that 4 shared an expression profile distinct from the other 8, in line with the division of cells in two major superclusters (delineated by dashed boxes, Supplementary Fig. 1d). Based on known markers of stromal cell types, these superclusters correspond to fibroblasts and mural cells (Supplementary Fig. 1e). This distinction in transcriptomic space underscores significant differences between the transcriptomes of fibroblasts and mural cells.

Within the fibroblast supercluster, we further classified a small subset of cells (cluster 9) as FAPs (Fig. 1c; Supplementary Fig. 1a). As described in our previous study^13^, FAPs are multipotent mesenchymal progenitors that give rise to SCA-1^−^ matrix-producing fibroblasts upon injury. These cells express elevated levels of markers indicative of progenitor-like characteristics, including *Ly6a* (SCA-1), *Pi16*, *Dpp4*, and *Anxa3*^22,23^, but not *Gli1* despite extensive reports of it being a marker of fibroblast progenitors^14,24,25^ (Supplementary Fig. 1f). In contrast, the *Ly6a*^low^ counterpart likely represents a more mature fibroblast state with increased expression of functional markers such as *Vcam1*, *Cxcl14*, *Apoe*, and *Col6a3*. *Vcam1*, in particular, has been regarded as a marker of pro-fibrotic fibroblasts in skeletal muscle^26,27^.

Mural cell clusters were regrouped into a VSMC subset and two pericyte subsets, herein annotated as PER-1 and PER-2 (Fig. 1c). Common gene markers associated with contractility, notably the expression of *Acta2*, *Myh11*, and *Tagln*^28,29^, were used to identify VSMC (Fig. 1d). Likewise, the pericyte subsets were distinguished using a set of established pericyte markers including *Kcnj8*, *Abcc9*, *Colec11*, and *Vtn*^28^. PER-2 is set apart from PER-1 given their separation in transcriptomic space. Indeed, we found the expression of *Cspg4* to be largely absent in PER-2. Further comparison between the pericyte subsets revealed that PER-2 displays a contractile program similar to that seen in VSMC (Fig. 1e). Additionally, it exhibits a fibrogenic program consisted of genes encoding for structural matrix proteins and matricellular factors typically found in fibroblasts. These overlapping signatures with other stromal subsets suggest that PER-2 may exhibit multiple functional roles. Interestingly, PER-2 also retains a unique signature consisted of genes related to immune cell recruitment (*Lgals3* and *Ccl11*) and vascular development (*Ccn3*, *Flt1*, and *Angpt2*), among others (Supplementary Fig. 1g). To localize this rare pericyte subset, we examined for galectin-3 (GAL-3; encoded by *Lgals3*) expressing mural cells in *Hic1*-CT2/tdTomato/*Pdgfra*-EGFP hearts (Supplementary Fig. 1h, top). While PER-2 did not appear to be confined to a particular anatomical region, co-staining with CD31 indicated their close association with small vessels (Supplementary Fig. 1h, bottom). Interrogation of relative proportions between the pericyte subsets by flow cytometry confirms the rare presence of PER-2 (Supplementary Fig. 1i). We noted that nestin (*Nes*) and GLAST (*Slc1a3*), previously described as markers differentiating subtypes of pericytes^30–32^, do not differ in expression across mural cell clusters. In fact, both genes appear to emphasize the distinction between fibroblasts and mural cells rather than identify different types of pericytes (Supplementary Fig. 1j).

Next, we sought to examine the regulatory programs governing each stromal subset. We applied Single-Cell rEgulatory Network Inference and Clustering (SCENIC) to reconstruct gene regulatory networks (GRNs) termed “regulons” and infer their activities in individual cells^33^. Each regulon is defined as a set of genes and a transcription factor (TF) predicted to regulate their expression. Hierarchical clustering with regulon activities confirms that mural cells exhibit a different set of regulatory programs from fibroblasts (Supplementary Fig. 2a). Specifically, many of the differentially active TFs in both fibroblast subsets (Fig. 1f) include those critical for fibroblast fate (*Tcf21* and *Twist2*)^34,35^, matrisome production (*Egr1*, *Nfkb1*, and *Sox9*)^19,36,37^, and regulation of fetal-specific programs (*Nfix*, *Nfic*, and *Nfia*)^38^. In contrast, the active regulons in mural cells are predominantly those involved in regulating angiogenesis (*Bach1*, *Ets1*, *Elk3*, *Mef2a*, *Mef2c*, *Meox2*, and *Yy1*; Fig. 1g)^39–43^. We noted that the transcripts of several of these TFs are expressed in both mural cells and fibroblasts at similar levels. However, their regulons are preferentially active in one but not the other, revealing additional layers of regulation in the maintenance of distinct cell identities. Variations in GRNs were also observed within mural cells and fibroblasts, albeit to a lesser degree.

Together, our results showed that mural cells are distinct from fibroblasts in the heart at homeostasis. Anatomically, mural cells, unlike the vast majority of fibroblasts, are embedded under the vascular basement membrane. This positional distinction is consistent with their differences in transcriptomes, and therefore impart unique biological functions in the heart stroma.

### Myocardial infarction induces cardiac pericyte activation

During MI, cardiac extracellular matrix (ECM) undergoes extensive remodeling where the loss of cardiomyocytes results in tissue displacement with a collagen-based scar^44^. Within the cardiac stroma, fibroblasts have been shown as the main source of matrix building blocks and matricellular factors PI. However, the role of mural cells in scar formation and, in general, their behavior in response to MI remains unclear^45^. Here, we assessed changes in the mural cell landscape following left anterior descending (LAD) coronary artery ligation, a murine model of MI.

**Fig. 2:**
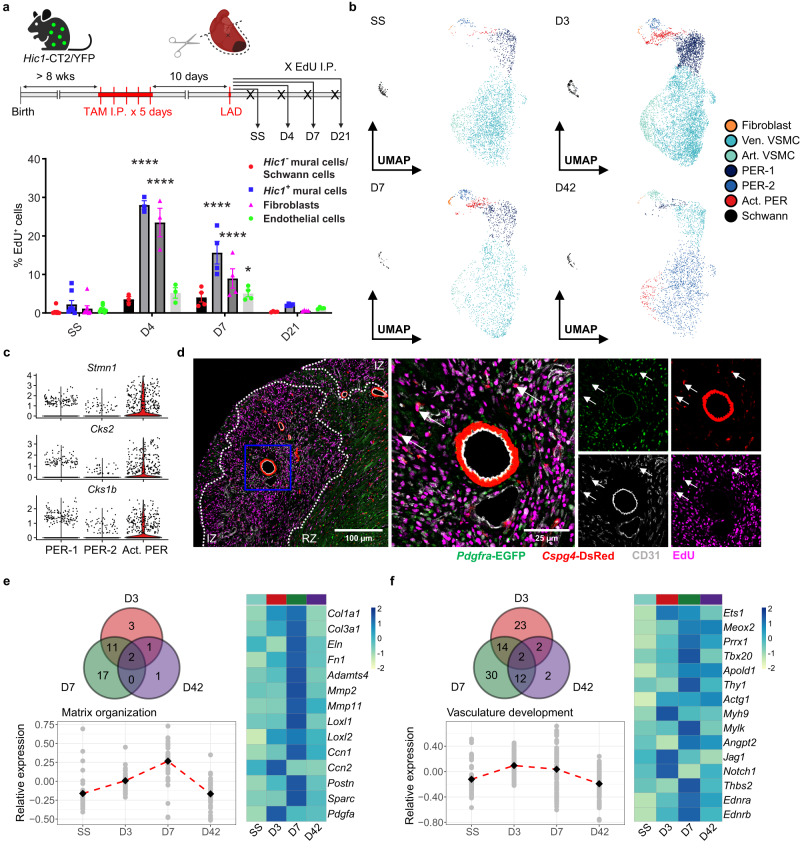
Pericytes adopt an altered state in MI. **a** Experimental scheme and assessment of cell proliferation by EdU incorporation on gated populations (*Hic1*^−^ mural cells/Schwann cells = LIN^−^YFP^−^CD146^+^, *Hic1*^+^ mural cells = LIN^−^YFP^+^CD146^+^, Fibroblasts = LIN^−^YFP^+^CD146^−^, Endothelial cells = LIN^+^YFP^−^CD146^+^) at steady state (SS) and day 4, 7, and 21 after LAD ligation (*n* = 8, 3, 4, and 4, respectively, pooled from ≥3 experiments). LIN = CD45 and CD31. Mice were administered with EdU IP one day before harvest. A two-way ANOVA with Dunnett’s post hoc test was used to compare the means of % EdU^+^ cells to SS in each respective gate. *P*-value: <0.0001, <0.0001, <0.0001, <0.0001, and 0.0395. Asterisks indicate statistically significant changes: *P* < 0.05 (*), *P* < 0.01 (**), *P* < 0.001 (***), *P* < 0.0001 (****). Data are presented as mean ± standard error of the mean. **b** UMAP projection of 15,355 LIN^−^CD146^+^ cardiac cells colored by subset annotations at SS and day 3, 7, and 42 PI. Ven. VSMC, venous/venular vascular smooth muscle cells; Art. VSMC, arterial/arteriolar vascular smooth muscle cells; PER, pericytes. **c** Normalized expression of cell cycle genes differentially upregulated in act. PER. **d** Immunofluorescence microscopy of the infarcted heart stained for CD31 (gray) and EdU (magenta) in *Pdgfra*-EGFP/*Cspg4*-DsRed (green/red) mice on day 7 PI (with EdU regimen starting at day 3). The infarct zone (IZ) is delineated from the remote zone (RZ) by dotted lines and a magnified inset (blue box) is provided to show EdU^+^ pericytes indicated by arrows. Scale bar = 100 µm or 25 µm (inset). Representative image of three biological replicates. **e**, **f** Assessment of temporally regulated DEGs associated with “ECM organization” (**e**) and **“**blood vessel development” (**f**) in pericytes. Venn diagrams represent the number of overlapping and unique DEGs mapped to each GO term across time points. Strip plots display the average scaled expression of DEGs with the means taken to generate the trend line. Heatmaps represent the relative average normalized expression of genes of interest. Source data are provided as a Source Data file.

To evaluate the proliferation kinetics of mural cells, we administered 5-ethynyl-2’-deoxyuridine (EdU) at multiple time points post-LAD surgery in *Hic1*-CT2/YFP mice (Fig. 2a). Heart ventricles were harvested and EdU incorporation was analyzed by flow cytometry (Supplementary Fig. 2b). We found that proliferation of *Hic1*-expressing mural cells (YFP^+^/CD146^+^/LIN^−^) peaks at day 4, at a level comparable to that of fibroblasts (YFP^+^/CD146^−^/LIN^−^). At day 7, the proliferation of both stromal populations is reduced and by day 21, it returns to steady state (SS) level. In the same timeframe, endothelial cells (YFP^−^/CD146^+^/LIN^+^) exhibit peak proliferation at day 7 PI, indicating active vessel formation in the infarcted heart. Interestingly, EdU incorporation in YFP^−^CD146^+^LIN^−^ cells is minimal. To pinpoint the identity of these cells, we sequenced CD146^+^ cells and compared the resulting cell landscape to that of *Hic1*-labeled cells. Our comparison demonstrated that *Hic1*-labeling excludes not only Schwann cells but also a subset of VSMCs expressing high levels of *Cnn1*, a marker of arterial and arteriolar VSMCs, herein collectively termed as art. VSMC^29^ (Supplementary Fig. 2c). Immunofluorescence staining with calponin 1 (CNN1) and alpha smooth muscle actin (αSMA) validated that CNN1 expression is exclusive to art. VSMC (Supplementary Fig. 2d). Furthermore, we did not observe co-localization between tdTomato and CNN1 signals in the vessel walls, confirming that the *Hic1* transgenic model does not trace all mural cells. Based on *Hic1*-labeling and CD39 (*Entpd1*) expression, which differentiates between VSMCs and pericytes at both the transcript and protein levels^46^ (Supplementary Fig. 2e, f), we were able to discern CD146^+^ populations as pericytes (tdTomato^+^CD39^−^), Schwann cells (tdTomato^−^CD39^−^), venous/venular VSMCs (ven. VSMC; tdTomato^+^CD39^+^), and art. VSMCs (tdTomato^−^CD39^+^). Using this gating strategy, the EdU incorporation assay was repeated at day 3 PI to evaluate proliferation in specific mural cell subsets, which showed that the majority of proliferating mural cells are pericytes (Supplementary Fig. 2g).

Next, we generated a scRNAseq time series to explore the transcriptional dynamics of mural cells. CD146^+^ cells were isolated from day 3, 7, and 42 PI and sequenced. Together with the uninjured dataset, we applied Seurat’s anchor-based integration across the four datasets, an approach used to harmonize datasets and minimize technical variation. Louvain clustering revealed that the annotated subsets we identified in *Hic1*-expressing mural cells were present at all time points PI (Fig. 2b). As we anticipated, sorting with CD146 independent of *Hic1*-labeling yielded a much more prominent population of art. VSMCs. Remarkably, we observed the emergence of an injury-induced mural cell cluster at day 3 PI (cluster 6, Supplementary Fig. 3a). Correlation analysis indicates substantial overlap in its transcriptome compared to those of the pericyte subsets (cluster 0 and 7, Supplementary Fig. 3b). In fact, many of the markers used to identify pericytes are co-expressed in cluster 6 (Supplementary Fig. 3c). Given these characteristics, we annotated this cluster of cells as “activated pericytes” (act. PER), reflecting its injury-responsive origin. Active proliferation in these cells is evident, as shown by the expression of mitotic genes (Fig. 2c). Follow-up assessment by histology indicated that cell division from day 3 to 7 PI, including that of *Cspg4*^+^ cells, is largely confined to the myocardial scar, and thus associates the localization of act. PER with the infarct (Fig. 2d). Differential expression does not reveal any gene marker exclusive to act. PER. However, many genes involved in matrix remodeling and vascular programs, as well as *Acta2*, are upregulated in this subset compared to the rest of pericytes irrespective of injury time point (Supplementary Fig. 3d). These changes point to a pathological response resembling that of fibroblast-to-myofibroblast transition. To validate the origin of act. PER, we repeated the sequencing experiment at day 7 PI by sorting CD146^+^ cells from the *Cspg4*-CT2/tdTomato transgenic line to specifically label *Cspg4*^*+*^ mural cells and their derivatives (Supplementary Fig. 3e). Much like the time series data, we captured the same activated subset in the mural cell compartment. This subset expresses a comparable level of *tdTomato* as other mural cells, corroborating its identity as an injury-induced progeny of *Cspg4*^*+*^ mural cells.

In addition to a discernible response in act. PER, temporal assessment of all pericytes indicated a global change in their transcriptional profile PI. Matrix-associated programs, encompassing matrix building blocks (*Col1a1*, *Col3a1*, *Eln*, and *Fn1*), metalloproteinases (*Adamts4*, *Mmp2*, and *Mmp11*), crosslink-inducing enzymes (*Loxl1* and *Loxl2*), and other matricellular factors regulating fibrosis (*Ccn1*, *Ccn2*, *Postn*, and *Sparc*), are activated PI (Fig. 2e). This aligns with the period during which fibroblasts expand and amplify their fibrotic response, suggesting that pericytes may exhibit an overlapping role in scar formation and collagen turnover. Interestingly, the upregulation of *Pdgfa*, a potent mitogen of fibroblasts, points to their potential role in inducing fibroblast expansion^47^. Furthermore, changes associated with regulators of vascular function were also observed (Fig. 2f). These include elevated expression of TFs (*Ets1*, *Meox2*, *Prrx1*, and *Tbx20*), cell surface proteins (*Apold1* and *Thy1*), cytoskeletal components (*Actg1*, *Myl9*, and *Mylk*), angiogenic elements (*Angpt2*, *Jag1*, *Notch1*, and *Thbs2*), and regulators of vascular tone (*Ednra* and *Ednrb*), reflecting an adaptive response in pericytes that affects vessel growth and stability. *Angpt2*, in particular, has been shown to mediate mural cell detachment from the vascular endothelium^48^. *Thbs2* may play a role in dampening pro-angiogenic mechanisms PI^49^. Notably, mediators of inflammation (e.g., *Cxcl1*, *Hmgb2*, *Il1r1*, and *Il6*) are also upregulated and therefore do not completely exclude pericytes’ role in inflammatory response (Supplementary Fig. 3f). Together, these injury-induced alterations in transcriptional programs are indicative of a shift in pericyte behavior toward a pathological state. While similar changes are seen in VSMCs, they are observed to a much lesser extent compared to pericytes (Supplementary Fig. 3g).

Collectively, it is evident that MI induces a significant transcriptional response in pericytes, while VSMCs appear less affected. This response is characterized by increased matrix remodeling and alterations in vascular programs. However, an activated subset associated with the infarct goes beyond this partial response and acquires a qualitatively similar but quantitatively distinct transcriptional program.

### Cardiac fibroblasts and mural cells do not share a lineage relationship in MI

Fibroblasts, specifically FAPs, are multipotent progenitors capable of differentiating into a number of cell types in response to injury. In arrhythmogenic cardiomyopathy, we had previously shown that cardiac FAPs can give rise to intramyocardial adipocytes^13^. Others have also described myocardial calcification as a consequence of fibroblasts adopting osteoblast-like properties^50^, supporting their multipotency. Nonetheless, whether FAPs or fibroblasts contribute to mural cells or vice versa remains elusive.

**Fig. 3:**
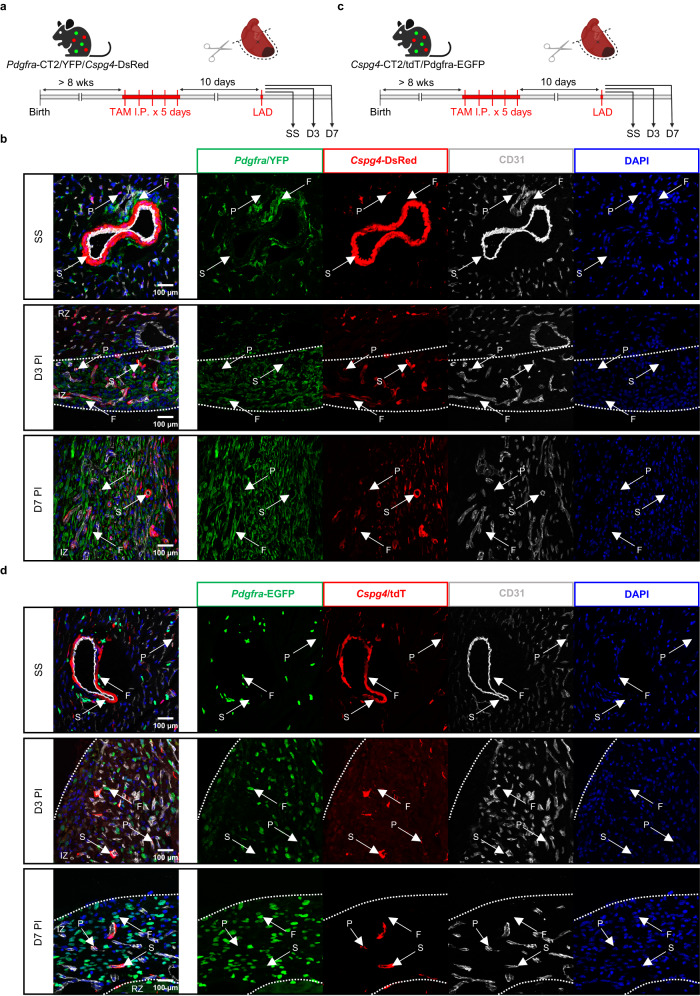
Fibroblasts and mural cells do not share a lineage relationship in MI. **a** Experimental scheme of lineage-tracing fibroblasts in MI. **b** Confocal images of labeled fibroblasts (YFP^+^, green) and mural cells (DsRed^+^, red) with respect to vascular endothelium (CD31, gray). Scale bar = 100 µm. Representative images of two to three biological replicates. **c** Experimental scheme of lineage-tracing mural cells in MI. **d** Confocal images of labeled mural cells (tdTomato^+^, red) and fibroblasts (EGFP^+^, green) with respect to vascular endothelium (CD31, gray). DAPI (blue) was used for nuclear counterstain. Scale bar = 100 µm. Representative images of one biological replicate. Arrows are indicative of VSMC (S), fibroblast (F), and pericyte (P). Dotted lines delineate the infarcted heart into remote (RZ) or infarct (IZ) zones.

Here, we adopted the *Pdgfra*-CT2/YFP mouse line to label cardiac fibroblasts at SS and follow their progeny at different time points post-LAD (Fig. 3a). Additionally, we crossed this mouse line with *Cspg4*-DsRed mice to generate a model that would accurately capture the acquisition of *Cspg4* expression in the lineage-traced progeny. Interrogation of YFP^+^ cells by immunofluorescence microscopy showed no co-localization events between YFP and DsRed signals in the infarct (Fig. 3b). In fact, YFP^+^ cells are interspersed throughout the infarct, whereas DsRed^+^ cells associate closely with endothelial cells as we had observed previously. Similarly, to address whether mural cells can give rise to fibroblasts, we generated a *Cspg4*-CT2/tdTomato mouse line that is crossed to the *Pdgfra*-EGFP reporter (Fig. 3c). After LAD ligation, tdTomato^+^ cells do not show overlapping signals with EGFP and remain in immediate proximity to endothelial cells in the infarct (Fig. 3d). This is supported by flow cytometry data where EGFP expression in the lineage-traced mural cells is largely negligible and unchanged throughout the course of injury (Supplementary Fig. 4a).

To substantiate these findings, we sequenced mural cells and fibroblasts using the *Hic1*-CT2/tdTomato system on day 7 PI and examined for changes in the gene expression of identity markers characteristic to either stromal cell types after MI (Supplementary Fig. 4b). Comparison of fibroblasts at SS and after injury did not show significant changes in the expression of mural cell-specific markers, including *Cspg4*, *Mcam*, and *Notch3* (Supplementary Fig. 4c). Their expression remained largely confined to mural cells when compared to fibroblasts after injury. Likewise, the expression of fibroblast-specific markers such as *Pdgfra*, *Cd34*, and *Lum* was minimal and unchanged in mural cells.

Together, our results strongly support that fibroblasts and mural cells do not share a lineage relationship in response to MI. Instead, both stromal populations can adopt an activated state, which may share a similar set of injury-induced programs.

### Progressive vascular rarefaction and hypertrophy in the infarct zone

**Fig. 4:**
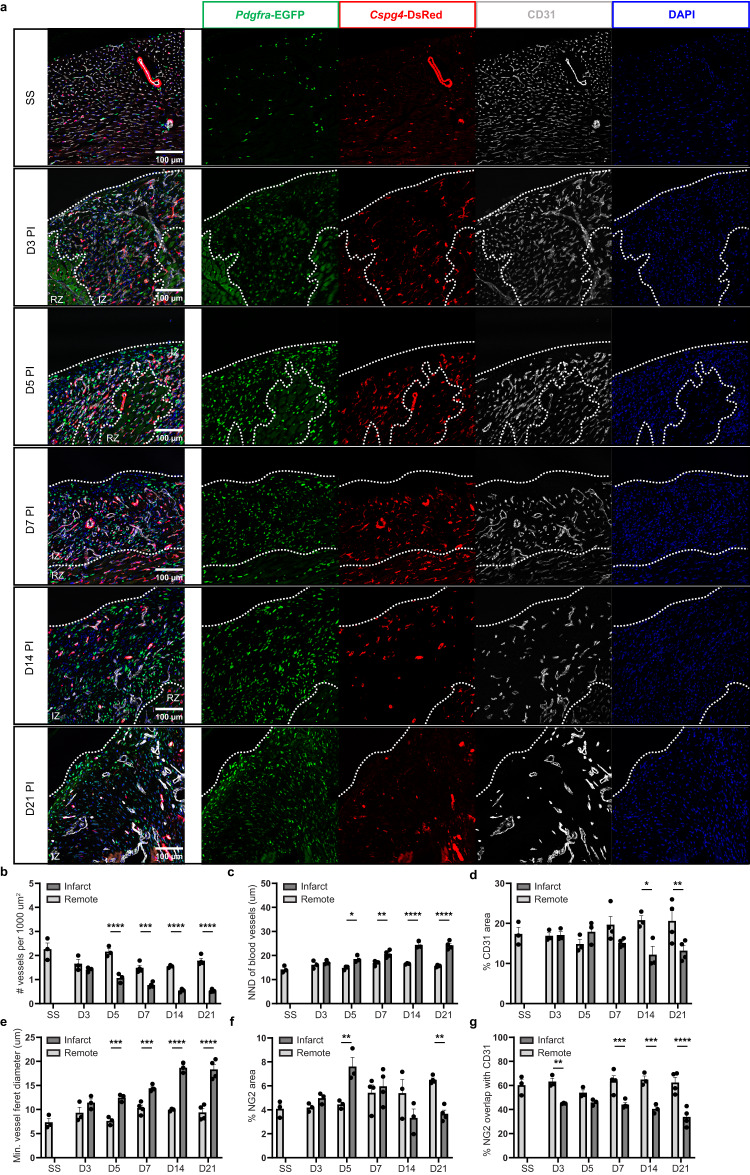
Progressive vascular deterioration in the infarct. **a** Immunofluorescence microscopy of the infarct zone at multiple time points PI. Heart sections from *Pdgfra*-EGFP/*Cspg4*-DsRed (green/red) mice were stained for CD31 (gray) and DAPI (blue). Dotted lines delineate boundaries between the remote (RZ) or infarct (IZ) zones. Scale bar = 100 µm. Representative images of three to four biological replicates. **b**–**g** Quantification and statistical testing of vascular parameters including: the number of vessels per 1000 μm^2^ (**b**; *P*-value: <0.0001, 0.0009, <0.0001, and <0.0001), nearest neighbor distance (NND) between vessels (**c**; *P*-value: 0.006, 0.0011, <0.0001, and <0.0001), proportion of CD31 area (**d**; *P*-value: 0.0112 and 0.0089), minimum average vessel feret diameter (**e**; *P*-value: 0.0008, 0.001, <0.0001, and <0.0001), proportion of NG2 area (**f**; *P*-value: 0.005 and 0.0036), and proportion of NG2 area overlapped with CD31 signals (**g**; *P*-value: 0.0078, 0.0008, 0.0003, and <0.0001). *n* = 3 (SS, D3, D5, and D14) or 4 (D7 and D21) pooled from 2 experiments. A two-way ANOVA with Šídák’s multiple comparisons post hoc test was used to compare the means of each parameter between the remote and infarct zones for every given time point. Data points derived from zones of the same sample are paired. Asterisks indicate statistically significant changes: *P* < 0.05 (*), *P* < 0.01 (**), *P* < 0.001 (***), *P* < 0.0001 (****). Data are presented as mean ± standard error of the mean. Source data are provided as a Source Data file.

Heart failure, including cases caused by MI, is closely associated with vascular dysfunction and rarefaction^51^. This, in part, has been attributed to the detachment and loss of mural cells, specifically pericytes, from microvasculature in the infarct^52^. However, the onset and progression of vascular impairment across the initial phases of tissue repair post-MI have not been investigated extensively. Moreover, it is unclear as to whether regions with distinct stromal compositions are established within the infarct over time. Here, we assessed changes in the vasculature and stroma by histological analyses using the *Pdgfra*-EGFP/*Cspg4*-DsRed line (Fig. 4a).

Co-staining of CD31 shows progressive deterioration of vasculature in the infarct zone. This is characterized by a drastic reduction in vascular density starting at day 5 PI (Fig. 4b), which is complemented with increasing nearest neighbor distance (NND) throughout the vascular network (Fig. 4c). Despite the loss of vessel density, we did not observe a significant decrease in the proportion of CD31 area until day 14 PI, suggesting that the difference may be masked by a shift in vessel size (Fig. 4d). Indeed, quantification of the minimum vessel feret diameter indicated increasing vascular hypertrophy from day 5 PI (Fig. 4e). It is also noted that vessels adopt a more misaligned and disorganized morphology over time. Through transcardiac perfusion of lectin-fluorophore conjugate, we found some of these vessels failed to be labeled by circulating lectin on day 14 PI, indicating a lack of blood perfusion and loss of vascular function (Supplementary Fig. 4d). In line with the EdU assay, mural cell expansion is reflected by a transient upsurge in the proportion of NG2 area on day 5 PI, which plateaus until a clear reduction is seen on day 21 PI (Fig. 4f). Interestingly, the proportion of NG2 area overlapping with CD31 shows an initial drop on day 3 PI, followed by a temporary recovery on day 5 PI and a continuous decline from day 7 PI onward (Fig. 4g). These data suggest that the initial impact from the infarction and inflammation cause mural cell detachment from the vascular endothelium. As the stroma undergoes a burst of expansion, nascent mural cells attempt to stabilize the vessels by re-associating with the vascular endothelium. Yet, this attempt is transient as mural cell proliferation ceases and likely becomes outpaced by cell death. Ultimately, the resulting vascular network at 3 weeks PI is sparse with limited coverage by mural cells.

After the initial expansion at day 3 PI, fibroblasts persist in elevated numbers and appear to be evenly distributed throughout the infarct. On the contrary, we have observed a two-layer organization within the infarct zone with respect to vascularization on day 5 and 7 PI in a subset of experimental mice (Supplementary Fig. 4e). This division is characterized by a vascularized inner layer and an outer layer that is solely populated by fibroblasts. Such organization points to a regional-specific response within the infarct whereby vessel loss is more prominent on the epicardial face than in regions closer to the endocardium. The lack of mural cells in the outer layer also substantiates their role in maintaining viable blood vessels.

Overall, our temporal analysis of the infarct indicates that vascular impairment is exacerbated with time post-MI. Although the proliferation of vascular cells points to active revascularization PI, the vascular network displays morphological abnormalities and remains much less permeated compared to that of the physiological state or remote zone. More importantly, there is a strong correlation between mural cell coverage and vascular dysfunction, whereby mural cell detachment precedes vessel loss.

### Spatiotemporal atlas of the infarcted heart

Single-cell transcriptomics offers insight into cellular mechanisms at single-cell resolution. However, the spatial information of cells is not preserved. In MI, the distribution of cells in the infarct zone and its surrounding regions is crucial to understanding the organization of the myocardial scar. More importantly, the identification of local processes and their underlying communication networks are vital for interpreting regional remodeling events, including inflammation, fibrosis, and vascular rarefaction. Here, we generated a time series of spatially resolved transcriptomic data using the 10x Genomics Visium platform. Transverse sections were cut from the hearts of *Pdgfra*-EGFP mice at SS and day 3, 7, and 14 PI. The resulting data contained 3852 uniquely barcoded “spots” or voxels, each of 55 µm in diameter, overlaid on the tissue sections.

**Fig. 5:**
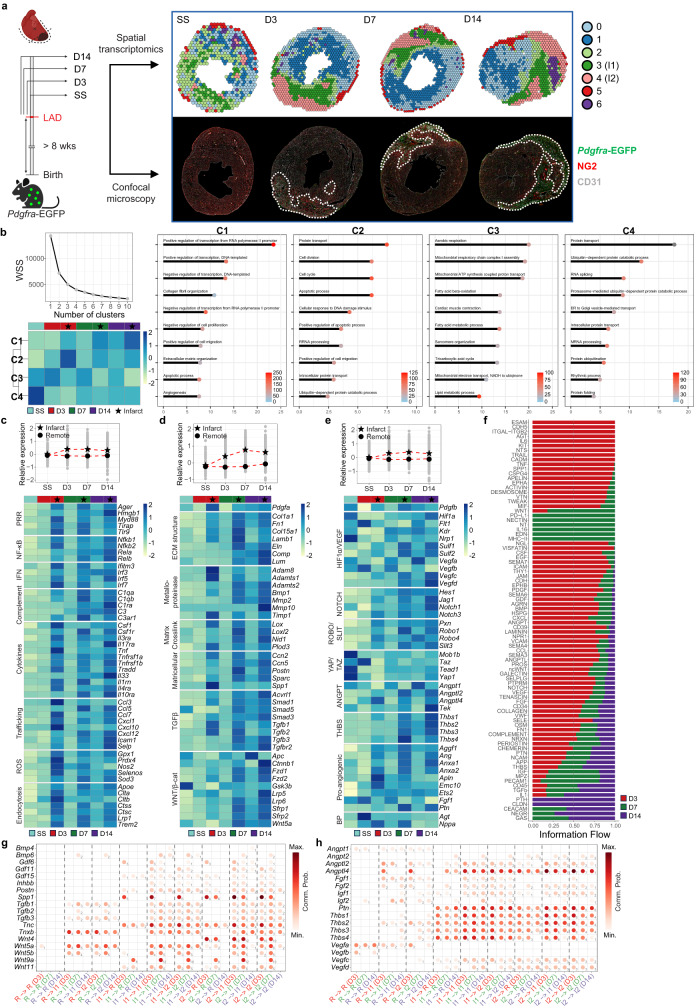
Spatiotemporal atlas of the infarcted heart. **a** Experimental scheme of generating the spatial transcriptomic time series. Voxels are colored by clusters after data integration across time points, with clusters 3 and 4 annotated as infarct-1 (I1) and infarct-2 (I2), respectively. Confocal images of serial sections complementing each dataset are presented and include stainings for NG2 (red) and CD31 (gray), along with endogenous *Pdgfra*-EGFP (green) signals. Dotted lines delineate the infarct zone. **b** K-means clustering of temporally regulated genes representing general patterns of gene expression. Elbow plot of within-cluster-sum of squared errors (WSS) against number of clusters indicated the inflection point at four clusters (C1-4). Heatmap depicts the relative average expression of cluster-specific genes per zone and time point. Segment-dot plots display the top 10 biological processes associated with each pattern. The length of segment and dot color intensity represent log-transformed adjusted *P*-values and the relative number of genes mapped to the pathway, respectively. **c**–**e** Assessment of temporally regulated genes associated with “inflammatory response” (**c**), “ECM organization” (**d**), and “blood vessel development” (**e**) per zone and time point. Strip plots display the average scaled expression of genes with means taken to generate the trend lines split by zone. Heatmaps represent the relative average normalized expression of genes of interest. **f** Relative information flow of all active pathways scaled by signaling strengths across time points. **g**, **h** Directionality and signaling strength of ligands associated with matrix remodeling (**g**) and vascular development (**h**). Dot color intensity reflects the sum of communication probabilities of each ligand, and the digit adjacent to each dot indicates the number of unique receptors associated with the ligand. Source data are provided as a Source Data file.

Louvain clustering of Visium spots elucidated regional differences in transcriptional programs (Fig. 5a). In the infarcted hearts, clusters are discerned into areas within the infarct and remote zones. Indeed, immunostaining on the same heart sections after library preparation shows the precise boundary of where viable myocardium meets the infarct based on autofluorescence (Supplementary Fig. 5a). Furthermore, the distribution of stromal cells and vasculature is elucidated by immunostaining with the respective serial sections. As expected, an increased density of fibroblasts and attenuation to the vascular network is evident in the infarct zone. Intriguingly, the infarct appears to be composed of two transcriptionally distinct layers at all time points PI: an inner layer (cluster 3; herein annotated as “infarct-1”) interfaced with the remote zone and an outer layer (cluster 4; annotated as “infarct-2”) with complete loss of cardiomyocytes. Broad assessment of gene signatures indicated that both regions of the infarct are characterized by elevated matrix, immune, and cell proliferation programs, as well as reduced gene expression in sarcomeric units (Supplementary Fig. 5b). However, infarct-1 exhibits a hybrid profile between infarct-2 and the remote zone, representing a region with intermediate remodeling programs that retains some residual cardiomyocytes. In contrast to the infarct zone, we did not capture significant changes among clusters in the remote zone or at steady state, and thus they are collectively annotated as “remote” or “SS” for further analysis.

To dissect the temporal response in the infarct and remote zones, we compiled a list of genes that are differentially expressed in at least one zone relative to SS for any given time point PI. These temporally regulated genes are clustered by k-means to identify gene sets with distinct expression patterns over time and across zones (Fig. 5b). Based on the clustering, we deduced four unique expression patterns and identified their top associated biological processes by pathway enrichment analysis. Cluster 1 (C1) is characterized by genes displaying progressive upregulation in the infarct zone with time. Its underlying processes are related to key tissue repair programs, including matrix remodeling and angiogenesis, and indicate changes to transcriptional regulation. Cluster 2 (C2) shows marked gene expression in the infarct zone on day 3 PI, followed by downregulation in subsequent time points but does not return to SS level. The associated pathways in C2 point to concurrent activation of cell division and apoptosis programs, which likely result from the expansion of stromal cells and cell death due to inflammation or ischemia. Genes in cluster 3 (C3) are progressively downregulated in the infarct zone and associate with processes required for maintaining energy metabolism and contractile function. Lastly, cluster 4 (C4) captures gene programs that are globally downregulated irrespective of zonation. Their associated pathways imply alterations in mechanisms governing gene expression, such as post-translational modification and RNA splicing, the efficiency of which has been reported to reduce in heart failure^53^.

In addition to examining holistic patterns of temporally regulated programs, we assessed signatures of known tissue repair processes underlying MI, specifically inflammatory response, matrix remodeling, and vascular development. In general, inflammation is increased in the infarct zone, whereas the remote zone remains largely unaffected PI (Fig. 5c). This increase corresponds to upregulated expression of pattern recognition receptors (PRR) and their downstream effectors, including NFκB and interferon regulatory factors. Moreover, complement cascade and cytokine signaling involving *Csf1*, *Tnf*, and interleukins are pronounced. Of note, *Il33*, a type-2 cytokine, peaks in expression at later time points, suggesting a potential shift from type-1 to type-2 inflammatory response. Likewise, chemokines and adhesion glycoproteins driving leukocyte recruitment are upregulated, along with programs related to oxidative stress regulation and endocytosis. Similar to the inflammatory response, the upregulation of matrix remodeling signatures is predominantly associated with the infarct zone (Fig. 5d). In parallel with the fibroblast kinetics, *Pdgfa* shows peak expression on day 3 PI and contributes to fibroblast expansion in the infarct during early stages of MI. With stroma activation, the production of matrix structural components, metalloproteinases, and enzymes that induce crosslinks to facilitate scar maturation is amplified. This remodeling process is supported by various matricellular factors and signaling cascades involving TGFβ and WNT/β-catenin pathways. It is noted that a minor increase in some inflammatory and matrix remodeling signatures is seen in the remote zone on day 14 PI. Whether this response reflects adaptive changes endemic to the remote zone induced by “spillover” of factors like alarmins from the infarct requires further investigation.

In terms of vascular development, there is an overall upregulation of its associated programs in the infarct zone (Fig. 5e). However, as a whole, it is not clear if these changes cultivate a tissue environment that reinforces or inhibits neovascularization in the infarct. The chemotactic factor of mural cells, *Pdgfb*, is downregulated in the remote zone on day 3 PI, which may create a gradient that promotes mural cell recruitment toward the infarct zone^54^. *Hif1a*, as a master switch upstream of many angiogenic factors, shows an initial drop in gene expression in both the remote and infarct zones but recovers by day 14 PI. Moreover, the well-established drivers of neovascularization, *Vegfa* and *Vegfb*, are downregulated at all time points in the infarct zone. Their cognate receptors also display transient downregulation at certain time points. On the other hand, the upregulation of sulfatases, *Sulf1* and *Sulf2*, may enhance VEGF signaling by increasing its bioavailability to the infarct^55^. Intriguingly, *Vegfc* and *Vegfd* demonstrate pronounced upregulation on day 7 and 14 PI, indicating a potential increase of lymphangiogenesis during the remodeling process. Genes involved in other major angiogenic pathways, including NOTCH and ROBO/SLIT signaling, show consistent upregulation in the infarct zone but not those of YAP/TAZ signaling. A number of secreted factors with pro-angiogenic properties like *Ang* are, too, upregulated with the exception of *Fgf1*. Comparably, we also observed apparent alterations in signals antagonizing neovascularization. For instance, members of the thrombospondin family, which confer anti-angiogenic effects, are jointly upregulated in the infarct zone. *Angpt1*, a factor known to promote vessel stability and maturation, is downregulated in the infarct, whereas its cognate receptor, *Tek*, exhibits the opposite pattern of expression. Interestingly, hormonal regulators of blood pressure such as *Agt* and *Nppa* display an early onset of expression in the remote zone and therefore represent another adaptive response to MI.

Collectively, these results indicate that tissue repair in MI is driven by regional-dependent and temporally regulated programs.

### Inter-regional communication networks regulate cardiac repair

Given the substantial differences in regional programs, we sought to recapitulate potential cellular interactomes underlying the annotated regions using a permutation-based approach^56^. From the inference, crosstalk among the infarct layers and remote zone is extensive with a significant proportion of active ligand-receptor interactions identified between and within the infarct layers (Supplementary Fig. 5c). The relative proportions of active interactions show that a subset of signaling pathways is exclusive to certain time points while others are approximately equal throughout the time series (Supplementary Fig. 5d). However, the relative information flow, defined as the sum of communication probability from all interactions under a signaling pathway, indicates that the strength of signaling is the highest on day 3 PI for the majority of pathways, reflecting a time point where regional communication is the most pronounced (Fig. 5f). To understand the “directionality” of information flow, we examined the “outgoing” (secretion of ligands) and incoming (reception of ligands) strengths of signaling pathways driven by secreted factors, specifically those involved in the tissue repair processes (Supplementary Fig. 5e).

Consistent with the upregulated expression of inflammatory signatures, signaling of various cytokine groups (CCL, OSM, IL, CSF, and TNF) is the most active in the infarct zone. These signals are largely sourced from within the infarct, with infarct-2 as the primary sender and receiver. Comparison of individual ligands reveals that a subset of interactions is only active in earlier time points and a decreasing trend of communication strength over time, indicating diminishing, but not complete termination of, inflammatory response (Supplementary Fig. 5f). Further analysis of interactions involving non-secreted factors shows that signaling of adhesion molecules for leukocyte diapedesis, such as *Icam-1*, *Icam-2*, and *Sele*, are limited to the infarct (Supplementary Fig. 5g).

Similarly, signaling groups known to associate with matrix production and remodeling (TGFb, BMP, GDF, WNT, POSTN, ACTIVIN, TENASCIN, and SPP1) are the most active in the infarct zone. While all ligands under these groups are sourced from the infarct layers, some are also derived from the remote zone, albeit at a reduced level of signaling strength compared to those local to the infarct (Fig. 5g). Additionally, the reception of these signals is present at reduced levels in the remote zone, which may contribute to reactive fibrosis in the uninjured myocardium. It is noted that certain signals display a stringent window of activation. For instance, signaling through *Bmp6*, a factor found to alleviate cardiac remodeling by attenuating fibroblast collagen production^57^, is limited to day 7 and 14 PI. Likewise, *Spp1* signaling is activated only in earlier time points, which may be critical for myofibroblast differentiation^58^. As expected, signaling through physical contact with ECM structural components, including collagens, laminins, and fibronectin, is restricted to the infarct.

Of the secreted signals that confer regulation of vascular development, the infarct zone is generally receptive to both inducers (VEGF, FGF, IGF, PTN, and ANGPT signaling via *Angpt2*) and inhibitors (ANGPTL, THBS, and ANGPT signaling via *Angpt1*) of neovascularization (Fig. 5h). However, the source of these angiogenic ligands can be regionally-specific. Indeed, *Vegfa* and *Vegfb* are exclusively sourced from the remote zone and act on both infarct layers across most time points. Interrogation by enzyme-linked immunosorbent assay (ELISA) indicated an overall increase of VEGFA in the heart at day 7 PI but no difference is seen between cardiac tissue proximal and distal to the infarct (Supplementary Fig. 5h). A similar signaling pattern is also exhibited by *Angpt1*, which has been shown to act in concert with the *Vegf* ligands to increase vascular density in the infarct zone^59^. Notably, the outgoing strength of FGF signaling is much higher in the remote zone and its reception in the infarct may pose as another pro-angiogenic mechanism^60^. On the contrary, the infarct zone is a dominant source of thrombospondins and *Angpt2*. *Thbs1*, in particular, has been implicated to induce impaired angiogenesis in MI^49^. Furthermore, its elevated expression is closely linked to the induction of *Angpt2*^61^, which antagonizes *Angpt1* signaling and exacerbates abnormal vascular remodeling. In addition to secreted signals, angiogenic signals requiring cell contact (NOTCH and Ephrins) are localized to the infarct layers. Of note, this included *Jag1* signaling, which induces vessel sprouting and tip cell formation during angiogenesis^62,63^. Yet, its pro-angiogenic effect may be counteracted by coinciding with *Dll4* activity.

Together, these differential patterns of regional communication suggest that, in response to MI, the remote zone acts as an important source of signals for stimulating neovascularization. While the infarct zone is receptive to these pro-angiogenic signals, it fails to maintain vascularization due to the presence of signals that attenuate vessel formation and integrity. Sustained inflammatory and pro-fibrotic signaling in the infarct may also contribute to vascular dysfunction. The resulting rarefaction, in part, is closely associated with the acquisition of a pathological state by pericytes and reduced mural cell coverage in the infarct. Indeed, localization of act. PER is best predicted to reside within the infarct layers on day 3 PI (Supplementary Fig. 5i). Our present data established a spatiotemporal atlas of the cardiac transcriptome and potential signaling networks underlying the tissue repair processes post-MI. These findings underscore the importance of regional and temporal considerations for the development of future therapeutics.

## Discussion

Cardiac stroma plays a critical role in tissue repair during myocardial ischemia. However, much of this regenerative process has been attributed to fibroblasts, given their role of forming a matrix scaffold, which is indispensable for sustaining cardiac function. On the contrary, mural cells were rarely in the spotlight despite their known involvement in facilitating reparative processes in other disease contexts such as spinal cord injuries^64^. Our study addressed this gap of knowledge by first examining the heterogeneity of mural cells and accentuating that they are distinct from FAPs/adventitial fibroblasts with a unique transcriptomic profile, regulatory programs, and localization pattern. Upon injury, we showed that pericytes undergo significant expansion and transcriptional alterations in matrix remodeling and vascular development programs. This, in part, is closely associated with progressive vascular rarefaction along with reduced perivascular coverage by mural cells. Lineage tracing of mural cells and fibroblasts also demonstrated a lack of lineage relationship between the stromal cell types in MI. By leveraging spatial transcriptomic assays, we uncovered an unappreciated spatiotemporal landscape of the infarcted heart and the communication networks underlying its tissue repair processes. In particular, we identified the remote zone as an important source of angiogenic factors for neovascularization. This is contrasted by an enriched production and reception of inflammatory, pro-fibrotic, and anti-angiogenic signals in the infarct zone. Overall, these data underpinned a correlation between vascular function and mural cell behavior and, more importantly, described regional-dependent mechanisms driving tissue repair PI. Our spatiotemporal atlas, specifically, is an invaluable resource for guiding potential avenues of improving therapeutic angiogenesis in MI.

A number of strategies have been used to profile stromal subsets in different organ systems. However, the lack of a consistent nomenclature has clouded our understanding of their identities, cellular functions, and lineage relationships. From our study, we demonstrated that the cardiac stroma is composed of non-overlapping cell types, fibroblasts and mural cells, using the double reporter system, *Pdgfra*-EGFP/*Cspg4*-DsRed. *Pdgfra*^13,65,66^ and *Cspg4*^67^ have been widely used as lineage reporters and transcriptomic markers for the identification of fibroblasts and mural cells, respectively. While this system is sufficient in distinguishing stromal subsets in the heart, the same cannot be said for other tissues such as the brain,^28,68^ and thus it is critical to evaluate reporter lines on a tissue-specific basis. At the transcriptomic level, it is clear that fibroblasts and mural cells retain specific sets of identity markers. However, we found that functional markers, such as *Col1a1*, *Angpt1*, and *Vegfa*, are not always confined to the cell type they have been traditionally associated with, especially after injury. This is partly due to some degree of redundancy in cellular functions, which may fluctuate depending on the environmental context. One example of this is the upregulation of contractile components (e.g., *Acta2*) in fibroblasts and, as we showed here, act. PER post-MI. As such, it is imperative to carefully consider the implications of using these functional markers as reporters, given the temporal and context-dependent restrictions of their utility. Further characterization of cells within the mural cell and fibroblast compartments revealed the presence of multiple subsets. Consistent with previous literature, fibroblasts can be classified into a progenitor-like subset (herein named “FAP”) and a more mature derivative (“fibroblasts”). Although mural cells are a more homogeneous population than fibroblasts^29^, we found considerable diversity in cardiac mural cells along the vascular axis. Specifically, we were able to distinguish those associated with arterial/arteriolar vessels (“art. VSMC”), venous/venular vessels (“ven. VSMC”), and capillaries (“PER-1” and “PER-2”). This level of diversity was rarely observed in previous studies as the yield of mural cells is typically low from tissue digestion. As mural cells are much smaller in proportion to other cardiac cells and more difficult to isolate given their long projections, future research examining mural cells should consider enrichment strategies using selective markers such as CD146.

Numerous studies have made claims about the presence of multiple pericyte subsets across a number of organ systems. Notably, Bribrair et al. identified 2 subtypes of pericytes based on Nestin-EGFP expression in lung, kidney, heart, and central nervous system (CNS)^30,31^. More recently, a similar characterization with GLAST (*Slc1a3*) expression was proposed to identify “type A pericytes” as the progenitor of scar-forming fibroblasts in the CNS^32^. From our data, the expression of either marker does not appear to differentiate between groups of pericytes. Rather, both genes are differentially expressed between the major stromal subsets where *Nes* is expressed throughout mural cells and *Slc1a3* in fibroblasts. Whether these discrepancies in pericyte characterization are attributed to true biological differences or inconsistency in nomenclature needs to be revisited in future studies. Here, we consistently identified two pericyte subsets with distinct patterns of expression in our baseline datasets: PER-1, which closely resembles canonical pericytes, and PER-2, which exhibits increased fibrogenic and contractile characteristics on top of a unique set of markers. Given its profile, it is likely that PER-2 corresponds to a rare cell state involved in physiological modifications of the perivascular matrix. Indeed, examination by histology indicated their association with microvasculature. Further research is required to characterize the biological function of this pericyte subset.

To date, the role of mural cells in cardiac injury and repair remains largely unknown. In fact, their proposed functions are mostly inferred from other organs or in vitro systems. This, in part, is due to the challenge of their identification in an injured environment where they adopt an altered morphology, expression profile, and location depending on the phase of tissue repair. In general, the stroma plays a multi-faceted role in response to acute injury. Immediately after the trauma, mural cells are speculated to participate in leukocyte trafficking by modifying vascular permeability and secreting cytokines, culminating in a pro-inflammatory environment that clears the wound of cell debris and matrix fragments. This is followed by the induction of activation signals to stimulate myofibroblast expansion, which amplifies matrisome production and the formation of a matrix scaffold that upholds cardiac integrity. From our study, we found that pericytes undergo significant expansion at a level comparable to that of fibroblasts on day 3 and 7 PI, whereas the proliferation of VSMCs is negligible. Temporal comparison of their transcriptome revealed that, collectively, pericytes adopt an injury-induced profile. This is characterized by a shift in programs associated with matrix remodeling, vascular development, and inflammation, some of which exhibit a transient window of upregulation. It is noted that this response is much more pronounced in a proliferative pericyte subset (“act. PER”) localized in the infarct. The increase in matrix signatures, in particular, suggests that pericytes may acquire some degree of functional redundancy with fibroblasts and contribute to scar formation in MI^69^. Importantly, this fibrogenic response is not associated with a lineage transition from or to fibroblasts, as shown by our lineage-tracing experiments using mural cell- and fibroblast-specific inducible systems. A similar finding in the context of diabetic fibrosis also demonstrated a lack of cardiac pericyte-to-fibroblast conversion^70^. However, these results contradict the findings of a recent study where a small fraction of infarct-associated pericytes has been shown to acquire *Pdgfra* expression^71^. Alterations in vascular programs may also point to the acquisition of a dysfunctional state that fails to provide adequate vascular maintenance. Despite the palpable adaptive response in mural cells, whether they are indispensable for scar formation or other tissue repair processes remains an area to be investigated.

One major hallmark in patients with heart failure is impaired vascular perfusion manifested in microcirculatory rarefaction and dysfunction. However, the processes underlying its onset are complex and likely result from perturbations in biochemical signals, biomechanical cues, and structural configurations. Inflammation, for instance, has been proposed as a key driver of endothelial dysregulation. Physiologically, vascular endothelium maintains a dilated state and exerts protective effects through preserving a fine-tuned bioavailability of nitric oxide (NO). This balance is lost during prolonged inflammation whereby pro-inflammatory cytokines such as TNFα reduce endothelial NO synthase (eNOS) expression and induce “eNOS uncoupling”, a state in which eNOS produces superoxides instead of NO due to the depletion of tetrahydrobiopterin^72^. The resulting surge in oxidative stress causes endothelial cell death through apoptotic programs like ferroptosis^73^. In our study, we found a significant decrease in the number of vessels in the infarct as early as day 5 PI. These compromised vessels exhibit morphological anomalies and, in some cases, are not perfused by blood circulation, which may be a consequence of the “no-reflow” phenomenon caused by mural cell hypercontraction. Moreover, we detected a drastic reduction in mural cell coverage around the vascular endothelium. Together, these results establish a convincing correlation whereby mural cell association with the vasculature is indispensable for preserving vascular function in MI. Indeed, given the role of mural cells in maintaining vascular quiescence and stability, their absence or detachment from the endothelial basement membrane has been associated with compromised vascular function in the kidney, brain, and, more recently, heart^74–76^. While we believe pathological signaling in the infarct leads to a dysfunctional state in mural cells, which in turn may induce detachment from the vasculature, the exact mechanism by which this occurs remains an important subject to be explored.

The potential to modify the ischemic scar and rebuild functional myocardium has been the primary focus of therapeutic intervention for IHD. Therapeutic angiogenesis, in particular, has gained traction as a strategy to repair myocardial injury and restore cardiac function. Indeed, an extensive body of research has established an irrefutable association between heart failure and functional impairment of the vascular network. By promoting cardiac perfusion, morphological and functional improvements in the infarcted heart have been observed. This is illustrated by a recent study in a rat model of MI where transplantation of human induced pluripotent stem cell-derived EC resulted in de novo formation of perfused vessels^77^. In turn, the increased vascular density in the infarct conferred cardioprotective effects as indicated by enhanced left ventricular function and reduced myocardial fibrosis. Comparably, pre-clinical investigations utilizing MSCs in cell-based therapies have also found a similar reparative response^78,79^. These effects are likely facilitated by paracrine signaling of factors such as VEGF and exosomes containing microRNAs (miRNA)^80^. Remarkably, the extent of clinical improvements can be enhanced by modifying MSCs prior to transplantation^81–83^. While cell-based therapies present a promising avenue for treatment, the effectiveness is inconsistent and often not sustainable long-term. This may be partly attributed to the poor engraftment and short retention of transplanted cells^84^. Moreover, different strategies of cell differentiation, isolation, and delivery may pose as a major confounder of therapeutic efficacy. Another important consideration is the location of transplantation relative to the infarct. Indeed, studies have reported regional differences in cell engraftment and regeneration^85^. In our spatial transcriptomic atlas, we have identified transcriptionally distinct regions in the infarcted heart: a remote zone with normal myocardium and an infarct core that can be subdivided into two layers (infarct-1 and infarct-2) with differing degrees of ischemia. Comparison between the remote and infarct zones revealed regional-dependent programs. While inflammatory and matrix remodeling signatures are focal to the infarct, signatures related to vascular development exhibit complex patterns that involve regulation by both the infarct and remote zones. This is further substantiated by regional communication networks underlying the infarcted heart. In general, signals propagating inflammation and fibrosis are largely produced and receptive within the infarct layers. On the other hand, the remote zone likely acts as an imperative source of angiogenic factors required for neovascularization. These differential signaling patterns underline the importance of “location” for localized treatments such as the application of acellular patches. In addition to positional considerations, the therapeutic efficacy is also contingent upon the timing of treatment. A meta-analysis of clinical trials indicated that left ventricular ejection fraction is much higher in patients who received MSC transplantation during the first week after MI^86^. In line with this conclusion, our data demonstrate that processes driving irreversible myocardial impairment may begin as early as day 3 PI. As such, the development of interventions for MI should focus on attenuating pathological mechanisms during the early stages of tissue remodeling.

Historically, promoting neovascularization has been the primary focus of therapeutic angiogenesis. However, the role of perivascular cells, especially mural cells, in vascular growth and regulation after injury is often overlooked. From the results of our study, deciphering the mechanisms behind mural cell activation, detachment, and, subsequently, loss will provide new angles to curtail vascular rarefaction and thus increase cardiac perfusion. For a multifocal disease like MI, these mechanisms are closely associated with the environment in which the mural cells reside. This, in turn, raises the prospect of improving the efficacy of cell-based therapies by concurrent modifications of regionally dependent programs in the infarcted heart.

## Methods

### Animals

Animal maintenance and experimental procedures were conducted in accordance with the Animal Care Committee’s approval and regulations at the University of British Columbia. Mice were housed under standard conditions (12:12 light-dark cycle, 21–23 °C, and 40–60% humidity level) and provided PicoLab Mouse Diet 20 (LabDiet, cat. 3005750-220) and water ad libitum in a pathogen-free facility. Non-inducible mouse lines, including PDGFRα^EGFP^ (referred to as “*Pdgfra*-EGFP”, JAX stock #007669) and NG2DsRedBAC (referred to as *Cspg4*-DsRed, JAX stock #008241) were purchased from The Jackson Laboratory. *Pdgfra*-EGFP mice were bred with *Cspg4*-DsRed mice to generate the double reporter mice, *Pdgfra*-EGFP/*Cspg4*-DsRed. Generation of TAM-inducible mouse lines harboring *Hic1*-CT2 is described elsewhere^13,19^. NG2-CreER^TM^ (referred to as “*Cspg4*-CT2”, JAX stock #008538) and Pdgfrα-CreER^T2^ (referred to as “Pdgfra-CT2”, JAX stock #032770) were purchased from The Jackson Laboratory. Each CT2 line was interbred with the reporter line B6.Cg-*Gt(ROSA)26Sor*^*tm14(CAG-*^^*tdTomato)Hze*^/J (referred to as “tdTomato”, JAX # 007914) or B6.129 × 1-*Gt(ROSA)26Sor*^*tm1(EYFP)Cos*^/J (referred to as “YFP”, JAX #006148). For lineage-tracing experiments, additional crosses were made with the non-inducible lines. Cre recombination was induced by intraperitoneal (IP) injections of TAM (Sigma-Aldrich, cat. T5648) at 3 mg/kg mouse weight in 100 µL of corn oil for 5 consecutive days. Mice administered with TAM were given at least 10 days of washout period prior to additional procedures. All experimental mice were maintained on a C57BL/6 background and ranged from 2 to 6 months in age. Littermates of both sexes were randomly assigned to different experimental groups wherever applicable. No blinding was performed and no data were excluded.

### Induction of cardiac injury

Experimental induction of myocardial infarctions was performed on animals ventilated with a mixture of oxygen and isoflurane until surgical anesthesia. The skin was surgically scrubbed with alcohol and Bupivacaine was administered subcutaneously at the incision site. An incision through the rib cage was made and held open with a retractor to visualize the heart. Using an 8-0 polypropylene suture, a branch of the left anterior descending (LAD) artery was ligated. The wound was then closed using a 6-0 vicryl suture in a horizontal mattress pattern^87^. Buprenorphine (20 µL/g) and saline (1 mL) were administered subcutaneously every 8 h as part of the monitoring procedure.

### Perfusion and tissue collection

Mice were euthanized with 0.5 mg/g tribromoethanol (Avertin; Sigma-Aldrich, cat. T4840-2) IP. Once euthanized, a horizontal incision was made above the sternum through both the skin and musculoskeletal layer to expose the heart. Following transcardiac perfusion with 20 mL of PBS-EDTA (2 mM), whole hearts were excised, and their atria were trimmed off. For immunofluorescence staining, heart samples were fixed in 4% PFA for 24 h at 4 °C, washed with cold PBS, and then incubated in 30% sucrose solution overnight. Samples were embedded into Optimal Cutting Temperature (OCT) compound (Tissue Tek, cat. 4583 or Scigen, cat. SGN4585) in plastic cryomolds and snap-frozen in an isopentane bath cooled by liquid nitrogen. For Visium experiments, heart samples were washed with cold PBS upon harvest and directly snap-frozen in OCT compound.

### Tissue digestion and purification of cell populations

Isolation of cells from cardiac ventricles was achieved using our previously established protocol^13^. After transcardiac perfusion, excised ventricles were cut into 2 mm pieces and digested in collagenase type II solution (Millipore Sigma, cat. C6885; 500 µL per heart at 2.5 U/mL) containing 5 mM CaCl_2_ for 30 min at 37 °C. The digested lysate was quenched with cold PBS and centrifuged at 140 × *g* to remove the supernatant. This process was repeated twice, followed by a second round of digestion in a solution (500 µL per heart) containing collagenase D (Millipore Sigma, cat. 11088882001; 1.5 U/mL), Dispase II (Millipore Sigma, cat. 04942078001; 2.4 U/mL) and 5 mM CaCl_2_ for 1 h at 37 °C. The digested lysate was triturated by pipetting, quenched with cold FACS buffer (PBS containing 2 mM EDTA and 2% FBS), and filtered through 40-μm strainer filters. Filtrate was centrifuged at 500 × *g* and washed again with cold FACS buffer prior to incubation with primary antibodies for 30 min at 4 °C. For all flow cytometric assays and cell sorting, anti-CD45 and anti-CD31 antibodies were used as lineage (LIN) markers to delineate immune and endothelial populations. To assess cell viability, live/dead staining was performed with fixable viability dye or a combination of propidium iodide, Hoechst, and DAPI. Flow cytometry and FACS were performed on BD Influx and Beckman Coulter CytoFLEX or LSR-II, respectively. Sorted cells were collected in DMEM (Gibco, cat. 11965-092) containing 20% fetal bovine serum (FBS; Sigma, cat. F1051). Flow cytometry data were analyzed using the software CytExpert (v.2.4, Beckman Coulter) or FlowJo (v.10.7.1, BD Biosciences). A table of primary antibodies used in the study is supplemented (Supplementary Data 1).

### Cell proliferation assay

Cell kinetics were assessed through the administration of EdU (Thermo Fisher, cat. E10415), followed by flow cytometry or immunofluorescence microscopy. For flow cytometry, mice were administered with EdU (20 mg/kg) dissolved in PBS (2 mg/mL) IP one day prior to harvest. Cells were stained for surface markers and EdU using the Click-iT Plus EdU Pacific Blue Flow Cytometry Assay Kit (Thermo Fisher, cat. C10418) as per the manufacturer’s directions. To assess the localization of proliferating cells between day 3 and 7 PI, we administered EdU IP on day 2 and 6 PI along with a continuous dose of EdU mixed in drinking water (5 mg/mL) until harvest. Immunostaining against EdU was performed using Click-iT™ Plus EdU Cell Proliferation Kit for Imaging (Thermo Fisher, cat. C10640) prior to antibody staining.

### Immunofluorescence staining

OCT-embedded tissues were sectioned using the Leica CM3050S cryostat at a thickness of 10-μm and placed onto Superfrost Plus slides (Fischerbrand, cat. 22-037-246). Slides were stored at −80 °C prior to thawing at room temperature for staining. Thawed sections were incubated in PBS containing 10 mg/mL sodium borohydride (Sigma, cat. 213462) for 30 min to quench autofluorescence and washed with PBS. Hydrophobic borders were drawn around tissue sections prior to incubation with a blocking buffer containing 5% normal donkey serum, 0.2% Triton X-100, and 1% bovine serum albumin (BSA; Sigma, cat. A7906) for 1 h at room temperature. Tissue sections were then incubated with primary antibody cocktails overnight at 4 °C, followed by incubation with secondary antibody cocktails for 1 h at room temperature. After each antibody incubation, tissue sections were washed with cold PBS three times for 5 min. Cell nuclei were counterstained with DAPI (600 nM). Slides were mounted with ProLong Gold antifade reagent (Invitrogen, cat. P36930) and stored at 4 °C. A table of primary and secondary antibodies used in the study is supplemented (Supplementary Data 1).

### Image acquisition

Confocal images were acquired using the confocal laser scanning microscope system LSM 900 with Airyscan 2 (Carl Zeiss) at 10X, 20X, or 40X magnification. The associated software for image acquisition is Zen Blue Edition (v.3.4, Carl Zeiss). Z-stack images were merged in ImageJ (v.1.53q) with Fiji via the method of maximum projection^88^. Scanning of large areas of interest was achieved by stitching multiple fields at 10X magnification. Merged and split channel images were exported and assembled in Adobe Illustrator 2021.

### Assessment of cardiac vascular network

To accurately characterize cardiac vasculature, imaging was completed using the same parameters with two technical replicates per sample. Areas were selected to capture continuous regions of the remote and infarct zones. Images shown in Fig. 4a depict representative regions focusing on the infarct core and were not used for quantification purposes. Image analysis was performed in ImageJ with a script adapted from a published workflow^89^. Initially, regions of interest (ROIs) were drawn to refine the boundaries of each zone and exclude empty gaps in the tissue section. This is followed by the conversion of individual channels, including CD31 and *Cspg4*-DsRed, to binarized images with the “Threshold” function. The threshold is set at a fixed value for all images stained and imaged in the same batch. Random noises attributed to abnormally bright pixels were minimized using the “Median” function under “Filter” with a radius parameter set at 2 pixels. Area fractions of CD31 and DsRed were then calculated separately as the percentage out of non-zero pixels within the defined ROIs. To evaluate perivascular mural coverage, we quantified the ratio of overlapping area between CD31 and DsRed to the total DsRed area. Based on CD31 signals, minimum Feret diameter and the number of distinct vessels above 1 μm^2^ in area were obtained from the “Analyze Particles” tab. The average nearest neighbor distance between vessels was computed using the “NND” plugin. Qualitative assessment of vascular blood flow was evaluated by transcardiac perfusion of lectin-fluorophore conjugate (Vector Laboratories, cat. DL-1178-1).

### ELISA

LAD heart ventricles were split into two parts: a “distal” segment above the sutures and another below that is “proximal” to the infarct. Tissues were snap-frozen and homogenized TissueLyser II (Qiagen, cat. 85300) in extraction buffer (Thermo Scientific, cat. 89900) mixed with Halt Protease and Phosphatase Inhibitor Cocktail (1:100; Thermo Scientific, cat. 78440). Protein concentration was determined using the Pierce bicinchoninic acid (BCA) Protein Assay Kit (Thermo Scientific, cat. 23225) as per the associated user manual. The amount of VEGFA present in the extracted protein was quantified using the ELISA kit (R&D Systems, cat. MMV00). All measurements were evaluated in duplicates.

### Single-cell library construction and sequencing

Cells from purified suspensions were counted using a hemocytometer and recombined to enrich target populations. Cell hashing with oligo-tagged antibodies against MHC class I and CD45 (BioLegend, cat. 155801/3/5/7) was applied to maximize cell yield and reduce cross-sample multiplets in samples corresponding to *Hic1*-expressing cells at steady state and CD146-sorted cells at 3 days PI. Libraries were generated using the Chromium Single Cell 3’ reagent kit (v.2 and v.3, 10X Genomics) as per the manufacturer’s protocol. Sample integrity was assessed on the Agilent Bioanalyzer 2100 system using the RNA 6000 Pico or Nano kit (Agilent, cat. 5067-1511 and 5067-1513). Sequencing was performed on an Illumina NextSeq 500 using the High Output 75 Cycle kit to a minimum depth of 30,000 mean reads per cell. Reads were demultiplexed and aligned to a custom mm10 reference genome with transgenic sequences, including tdTomato, YFP, and DsRed, using the Cellranger pipeline (v.4.0.0, 10X Genomics). The resulting feature-barcode matrices were imported into R (v.3.6.1) for downstream processing and analyses.

### Single-cell RNA-seq analysis

Data processing and differential expression analysis were performed using Seurat (v.3.2.0)^90^. Wherever applicable, demultiplexing with hashtag oligos (thresholding at 0.99 quantile) was applied to remove cross-sample multiplets. To remove cells of poor quality, metrics including the number of genes expressed (>200), total unique molecular identifier (UMI; <7000–15,000), and proportion of mitochondrial transcripts (<10–15%) per cell were tailored to each dataset. Log normalization was applied to UMIs, and the top 2000 variable genes were selected by variance-stabilizing transformation. The proportion of mitochondrial transcripts was regressed out to minimize clustering by cell quality. Anchor-based data integration was applied to appropriate batches based on experimental dates. The number of principal components (PCs) used for Louvain clustering and uniform manifold approximation and projection (UMAP) was determined by the inflection point of variances accounted for by PCs (~20 PCs). Clusters were grouped into distinct annotated subsets as advised by pair-wise Pearson’s correlation across all genes, as well as the expression of identity markers reported in past literature. Differential expression analysis was performed using the Wilcoxon rank sum test with *P*-values adjusted by Bonferroni’s correction. Only genes that were expressed in at least 25% of cells in one of the comparison groups and had a log2-fold change of at least 0.25 were considered. Temporally regulated genes were compiled by iterative differential expression comparisons of each injury time point to SS. Scaled expression was averaged to generate strip plots and the associated trend lines. Heatmaps of average normalized expression were generated using pheatmap (v.1.0.12) with scaling applied to each gene.

### Gene regulatory network analysis

Single-cell regulatory network inference and clustering (SCENIC; v.1.1.2-2) was applied to scRNAseq data to infer the activity of GRNs in individual cells^33^. Raw feature-count matrices were QC’ed in Seurat prior to analysis using the recommended settings according to the published vignette (https://github.com/aertslab/SCENIC) and the mm9 Rcistarget reference. Regulon activity scores were appended to the clustered Seurat objects and evaluated on the same UMAP embeddings. Hierarchical clustering was performed on binarized regulon activity scores to assess the regulatory programs unique to each annotated cell population. Networks of cell-specific regulons were drawn according to the inferred regulon targets. Only transcription factors active in at least 50% of any annotated population were considered in the analysis.

### Visium

Spatial transcriptomics of the infarcted heart was evaluated using the Visium Spatial Gene Expression platform (v2, 10X Genomics). Frozen heart samples were sectioned at 10-μm thickness and mounted onto a pre-cooled (−20 °C) glass slide. Note that serial sections were kept for each heart tissue used to generate positional transcriptomic datasets for immunostaining. Sections were fixed in cold methanol prior to H&E staining and imaged on brightfield with a Nikon Eclipse Ni microscope using the NIS Elements software (v.5.11.03, Nikon) as instructed in the Visium CytAssist demonstrated protocol (CG000614). Processing of tissue sections was performed in accordance with the Visium CytAssist user guide (CG000495) using the Visium CytAssist Spatial Gene Expression Kit (PN-100051). cDNA libraries were sequenced on a NextSeq 2000 sequencer (Illumina) with the P2 100 cycles reagents (Illumina, cat. 20046812) to a minimum depth of 40,000 mean reads per spot. Reads were aligned to the mm10 genome reference with Space Ranger (v.2.0.1, 10X Genomics) to generate positional feature-barcode matrices. Downstream processing with Seurat was completed as described in the spatial data vignette using SCTransform^91^. Integration across time points was applied to identify spots of similar expression profiles. Note that the apparent presence of spots from clusters 3 and 4 at SS is likely due to technical limitations of integration. Follow-up analysis shows that these spots do not resemble those of the “true” infarct zone after MI and do not demarcate areas of myocardial loss or fibrosis. Differential expression analysis was performed as described in “Single-cell RNA-seq analysis” with the SCT assay. K-means clustering using the average expression of temporally regulated genes was implemented to categorize genes of distinct patterns and identify their associated functions. The optimal number of clusters was estimated with the inflection point of within-cluster-sum of squared errors (WSS) plotted against clusters. To deconvolve the identity of voxels, data integration was applied using scRNAseq data as a reference to transfer cell type annotations and compute their prediction scores per spot.

### Pathway enrichment analysis

Pathway enrichment analysis was performed using DAVID Bioinformatics Resources (2023 Update)^92,93^ and results were extracted from under the “GO Direct” category. *P*-values were adjusted by Benjamini-Hochberg correction and log-transformed for plotting. Values adjacent to bars indicate the number of DEGs annotated in each GO term. To score the activity of general biological processes, the average expression of DEGs found in the Gene Ontology terms “inflammatory response” (GO: 0006954), “ECM organization” (GO:0030198), and “blood vessel development” (GO:0001568) was calculated per time point.

### Inference of regional communication networks

Communication networks of the annotated Visium data were assessed using CellChat^94^ (v.1.6.1) in accordance with the published vignette (https://github.com/sqjin/CellChat) in R (v. 4.1.2). Briefly, the integrated Seurat object was directly imported to create CellChat objects split by time point. All ligand-receptor interactions were queried from the CellChatDB database for *Mus musculus*. Over-expressed ligands and receptors across annotated regions were identified with *P*-value threshold set at 0.1 and passed to communication assessment. Communication probability was calculated with distance constraints whereby interactions beyond 5 voxels (~275 μm) were excluded. The strength of communication was normalized to the abundance of regional voxels during the probability calculation. Weak interactions with less than 10 voxels expressing the ligand or receptor in each region were filtered out. Pathways active in at least one time point were considered for presentation. Active interactions (i.e., interactions with *P*-value < 0.05) related to inflammation, matrix remodeling, and vascular development were manually selected and their signaling strengths were presented as a sum of all communication probabilities for each ligand. The digit adjacent to dots in dot plots represents the number of unique receptors that interact with the ligand.

### Statistical analysis

Statistical testing and graphing of data associated with flow cytometry, immunofluorescence microscopy, and ELISA were performed using Prism 9 (GraphPad Software). Error bars represent deviation by standard error of the mean (Mean ± SEM). The type of test, sample size, and adjusted *P*-values of each experiment are provided in respective figure legends. *P*-value < 0.05 was considered statistically significant. Degrees of significance are denoted as follows: *P* < 0.05 (*), *P* < 0.01 (**), *P* < 0.001 (***), *P* < 0.0001 (****). Figure assembly was performed in Adobe Illustrator 2021.

### Reporting summary

Further information on research design is available in the Nature Portfolio Reporting Summary linked to this article.

### Supplementary information

Supplementary Information

Description of Additional Supplementary Files

Supplementary Data 1

Reporting Summary

### Source data

Source Data

## Data Availability

The single-cell and spatial transcriptomic data generated in this study have been deposited in the Gene Expression Omnibus (GEO) database under the accession code GSE206787. All databases associated with the software and packages used in the study are described in the “Methods” section. To promote readership, the processed data can be accessed and probed on our publicly accessible platform, Integrated Single-cell NAvigation Portal (ISNAP) [https://isnap.rossilab.dev/GSE206787/]. Source data are provided with this paper.

## Code availability

All data processing and analysis scripts are constructed as per the vignettes of their respective sources and accessible on the GitHub repository [https://github.com/rossilab/spatial_temporal_atlas]. Please refer to “Methods” above for details of key parameters and package versions.
