# Supplementary material for: Spatiotemporal signaling underlies progressive vascular rarefaction in myocardial infarction
Source: Nat Commun. 2023 Dec 21;14:8498. doi: 10.1038/s41467-023-44227-6 (PMC10739910; doi:10.1038/s41467-023-44227-6)
Supplement: Supplementary file 1 — Supplementary Information [file 41467_2023_44227_MOESM1_ESM.pdf]

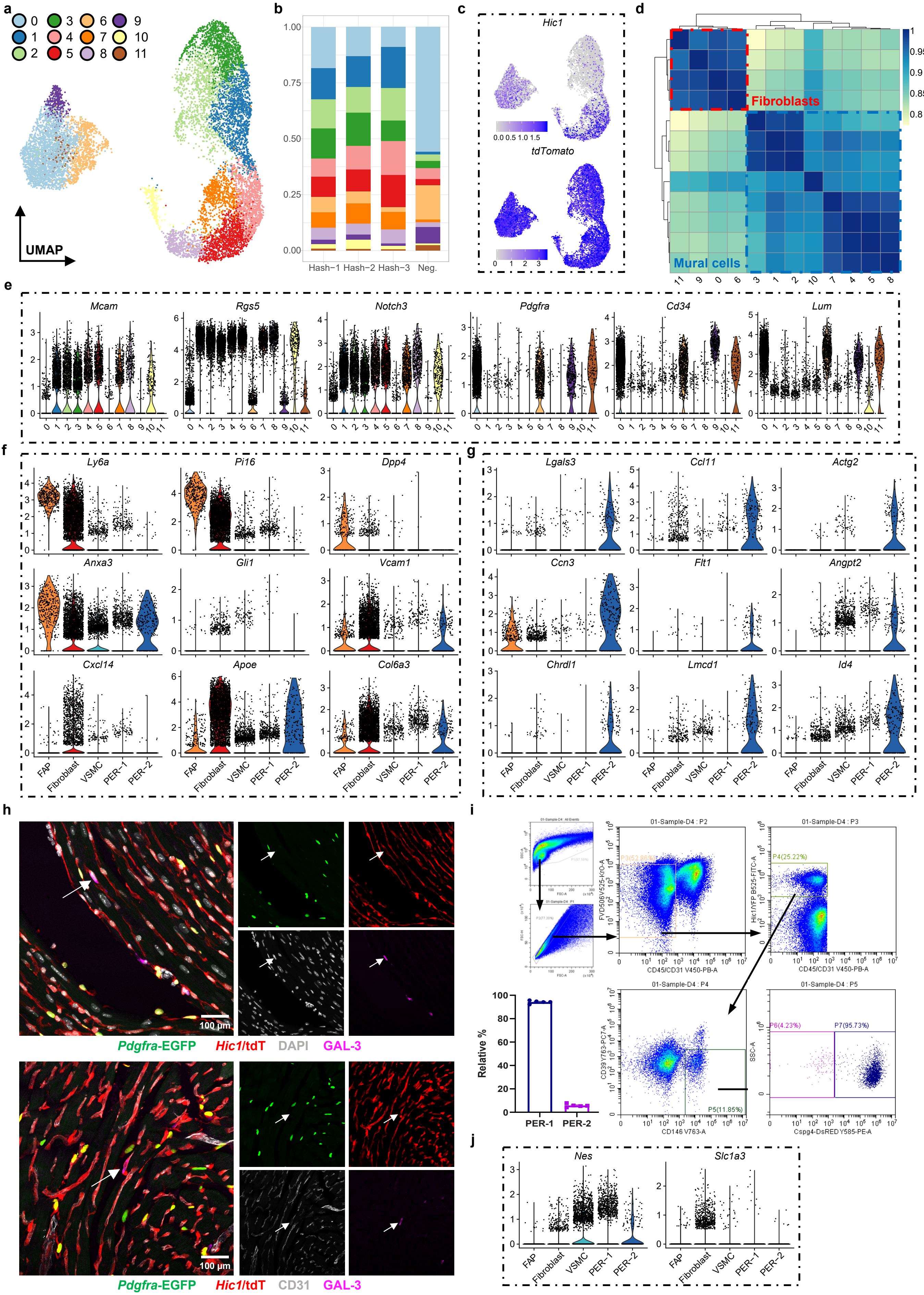
a
b
c
d
e
f
g
h
i
j
UMAP
Hash−1
Hash−2
Hash−3
Neg.
Hic1
tdTomato
Fibroblasts
Mural cells
Mcam
Rgs5
Notch3
Pdgfra
Cd34
Lum
Ly6a
Pi16
Dpp4
Anxa3
Gli1
Vcam1
Cxcl14
Apoe
Col6a3
Lgals3
Ccl11
Actg2
Ccn3
Flt1
Angpt2
Chrdl1
Lmcd1
Id4
FAP
Fibroblast
VSMC
PER−1
PER−2
100 µm
Pdgfra-EGFP
Hic1/tdT
DAPI
GAL-3
CD31
01-Sample-D4 : P2
01-Sample-D4 : P3
01-Sample-D4 : P4
01-Sample-D4 : P5
P4(25.22%)
P5(11.85%)
P6(4.23%)
P7(95.73%)
FVD506 V525-KrO-A
CD45/CD31 V450-PB-A
Hic1/YFP B525-FITC-A
CD39 Y763-PC7-A
CD146 V763-A
SSC-A
Cspg4-DsRED Y585-PE-A
Relative %
PER-1
PER-2
Nes
Slc1a3

Supp Fig 1. Characterization of cardiac stromal populations. a-b, UMAP projection of 11,489 tdTomato^{+} stromal cells colored by clusters (**a**) and their distribution from each hashed sample and non-hashed "negative" cells (**b**). Each hash represents an individual biological replicate (n = 3) pooled for library preparation and sequencing. **c**, Projection of normalized expression of *Hic1* and *tdTomato* on the UMAP. **d**, Pearson's correlation analysis showing the similarity of global transcriptional profiles across clusters. Dashed boxes delineate two major superclusters: fibroblasts (red) and mural cells (blue). Heatmap illustrates the relative magnitude of correlation coefficient. **e-g**, Normalized expression of known mural cell and fibroblast markers in clusters (**e**), reported progenitor and mature fibroblast markers (**f**), and markers unique to PER-2 (**g**) in annotated subsets. FAP, fibro-adipogenic progenitors; VSMC, vascular smooth muscle cells; PER, pericytes**. h**, Immunofluorescence staining with GAL-3 (magenta) and DAPI (grey, top) or CD31 (grey, bottom) in *Hic1*-CT2/tdTomato/*Pdgfra*-EGFP (red/green) hearts. Cells highlighted by arrows indicate GAL-3 expressing perivascular cells labelled by tdTomato (red) but not EGFP (green). Scale bar = 100 µm. Representative images of two biological replicates. PER, pericytes. **i**, Relative proportions of PER-1 ("P7") and PER-2 ("P6") out of all *Hic1*-labelled pericytes (n = 5). Representative gating strategy of all biological replicates. Data are presented as mean ± standard error of the mean. **j**, Normalized expression of *Nes* and *Slc1a3*. Source data are provided as a Source Data file.

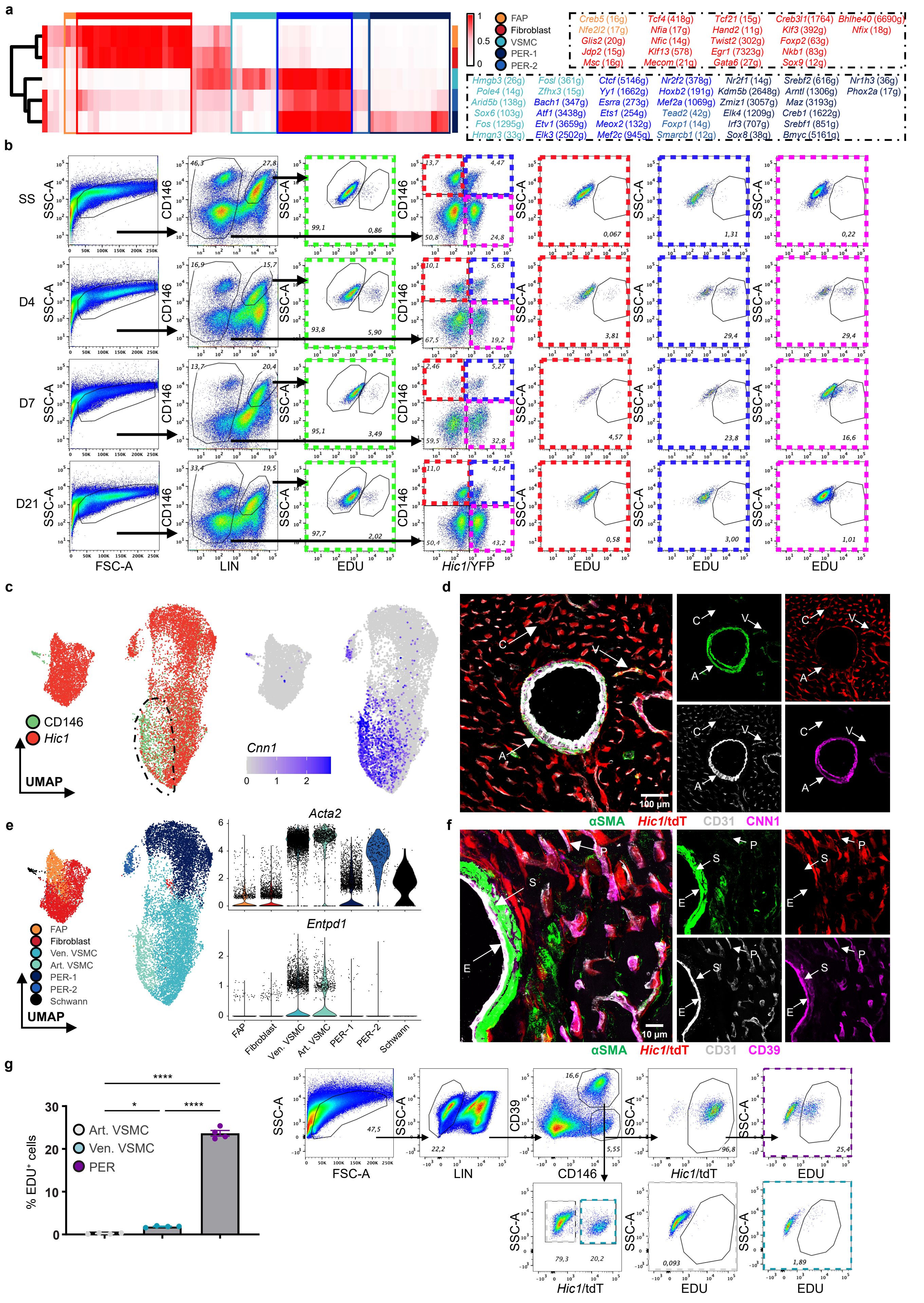
a
FAP
Fibroblast
VSMC
PER-1
PER-2
Creb5 (16g) Nfe2l2 (17g)
Tcf4 (418g) Nfia (17g) Nfic (14g) Klf13 (578) Mecom (21g)
Glis2 (20g) Jdp2 (15g) Msc (16g)
Tcf21 (15g) Hand2 (11g) Twist2 (302g) Egr1 (7323g) Gata6 (27g)
Creb3l1 (1764) Klf3 (392g) Foxp2 (63g) Nkb1 (83g) Sox9 (12g)
Bhlhe40 (6690g) Nfix (18g)
Hmgb3 (26g) Pole4 (14g) Arid5b (138g) Sox6 (103g) Fos (1295g) Hmgn3 (33g)
Fosl (361g) Zfhx3 (15g) Bach1 (347g) Atf1 (3438g) Etv1 (3659g) Elk3 (2502g)
Ctcf (5146g) Yy1 (1662g) Esrra (273g) Ets1 (254g) Meox2 (132g) Mef2c (945g)
Nr2f2 (378g) Hoxb2 (191g) Mef2a (1069g) Tead2 (42g) Foxp1 (14g) Smarcb1 (12g)
Nr2f1 (14g) Kdm5b (2648g) Zmiz1 (3057g) Elk4 (1209g) Irf3 (707g) Sox8 (38g)
Srebf2 (616g) Arntl (1306g) Maz (3193g) Creb1 (1622g) Srebf1 (851g) Bmyc (5161g)
Nr1h3 (36g) Phox2a (17g)
b
SS
D4
D7
D21
FSC-A
LIN
EDU
Hic1/YFP
EDU
EDU
EDU
c
CD146
Hic1
UMAP
Cnn1
d
C
V
A
100 µm
αSMA Hic1/tdT CD31 CNN1
e
FAP
Fibroblast
Ven. VSMC
Art. VSMC
PER-1
PER-2
Schwann
UMAP
Acta2
Entpd1
f
P
S
E
10 µm
αSMA Hic1/tdT CD31 CD39
g
% EDU⁺ cells
Art. VSMC
Ven. VSMC
PER
FSC-A
LIN
CD146
Hic1/tdT
EDU
CD39

Supp Fig 2. GRN signatures of stromal subsets at SS and cell kinetics after MI. a, Hierarchical clustering of inferred gene regulatory network (GRN) activities from SCENIC and lists of active transcription factors (TF) unique to fibroblasts (top) and mural cells (bottom) coloured by their associated subsets. Heatmap reflects the proportion of cells active for a particular TF in given a subset. Bracketed numbers indicate the number of downstream targets inferred to be regulated by the TF. FAP, fibro-adipogenic progenitors; VSMC, vascular smooth muscle cells; PER, pericytes. **b**, Representative gating strategy of cell subsets and their EdU-incorporated fractions PI. Gates are coloured by subset annotations corresponding to Fig 2a. LIN = CD45 and CD31. **c**, UMAP projection of cell transcriptomes between *Hic1*-labelled (red) and $CD146^+$ (green) cells (left). Projection of normalized expression of *Cnn1* on the UMAP (right). **d**, Immunofluorescence staining of αSMA (green), CD31 (grey), and CNN1 (magenta) in *Hic1*-CT2/tdTomato (red) hearts. Arrows indicate vessel types: capillary (C), artery (A), and vein (V). Scale bar = 100 μm. Representative image of three biological replicates. **e**, UMAP projection of integrated cell transcriptomes between *Hic1*-labelled and $CD146^+$ cells coloured by annotated subsets (left). Violin plots show normalized *Acta2* and *Entpd1* expression (right). Ven. VSMC, venous/venular vascular smooth muscle cells; Art. VSMC, arterial/arteriolar vascular smooth muscle cells; PER, pericytes. **f**, Immunofluorescence staining of αSMA (green), CD31 (grey), and CD39 (magenta) in *Hic1*-CT2/tdTomato (red) hearts. Arrows are indicative of VSMC (S), endothelial cells (E), and pericyte (P). Scale bar = 10 μm. Representative image of three biological replicates. **g**, Assessment of EdU incorporation in mural cell subsets on day 3 PI (n = 3). A one-way ANOVA with Tukey's multiple comparisons *post-hoc* test was used to compare the means of % EdU^+ cells. Representative gating strategy of all biological replicates. P-value: <0.0001, 0.0396, and <0.0001. LIN = CD45 and CD31. Asterisks indicate statistically significant changes: P < 0.05 (*), P < 0.01 (**), P < 0.001 (***), P < 0.0001 (****). Data are presented as mean ± standard error of the mean. Source data are provided as a Source Data file.

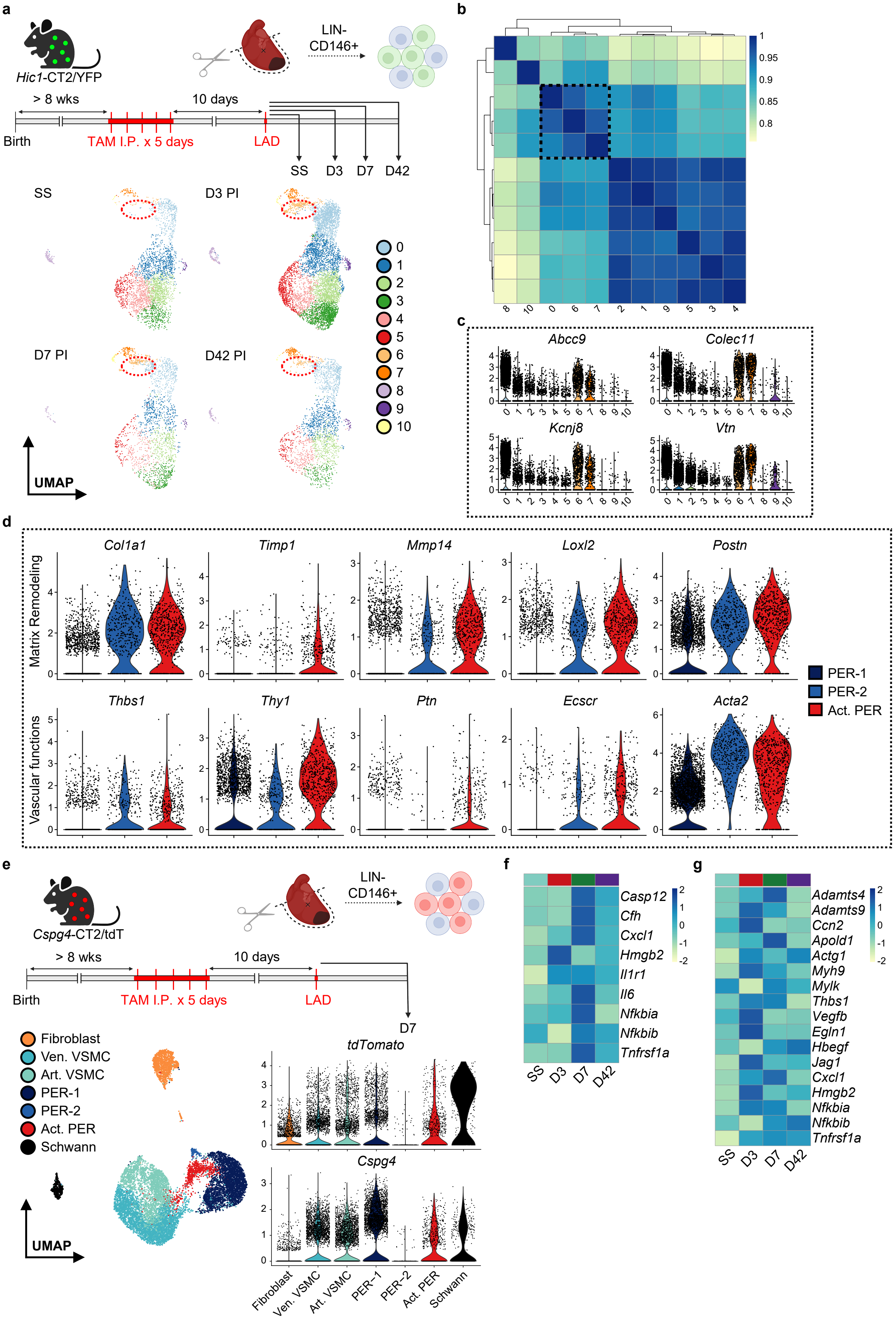
a
Hic1-CT2/YFP
LIN-
CD146+
> 8 wks
10 days
Birth
TAM I.P. x 5 days
LAD
SS
D3
D7
D42
SS
D3 PI
D7 PI
D42 PI
0
1
2
3
4
5
6
7
8
9
10
UMAP
b
1
0.95
0.9
0.85
0.8
8
10
0
6
7
2
1
9
5
3
4
c
Abcc9
Colec11
Kcnj8
Vtn
d
Matrix Remodeling
Col1a1
Timp1
Mmp14
Loxl2
Postn
Vascular functions
Thbs1
Thy1
Ptn
Ecscr
Acta2
PER-1
PER-2
Act. PER
e
Cspg4-CT2/tdT
LIN-
CD146+
> 8 wks
10 days
Birth
TAM I.P. x 5 days
LAD
D7
Fibroblast
Ven. VSMC
Art. VSMC
PER-1
PER-2
Act. PER
Schwann
UMAP
tdTomato
Cspg4
Fibroblast
Ven. VSMC
Art. VSMC
PER-1
PER-2
Act. PER
Schwann
f
Casp12
Cfh
Cxcl1
Hmgb2
Il1r1
Il6
Nfkbia
Nfkbib
Tnfrsf1a
SS
D3
D7
D42
g
Adamts4
Adamts9
Ccn2
Apold1
Actg1
Myh9
Mylk
Thbs1
Vegfb
Egln1
Hbegf
Jag1
Cxcl1
Hmgb2
Nfkbia
Nfkbib
Tnfrsf1a
SS
D3
D7
D42

Supp Fig 3. Mural cell response to MI. a, Experimental scheme and UMAP projection of 15,355 LIN$^-$CD146$^+$ cardiac cells from *Hic1*-CT2/YFP mice coloured by clusters at SS and day 3, 7, and 42 PI. Libraries were integrated to preserve cells of similar biological states. LIN = CD45 and CD31. **b**, Assessment of cluster similarities by Pearson's correlation analysis. Dashed box highlights pericyte clusters. Heatmap illustrates the relative magnitude of correlation coefficient. **c-d**, Normalized expression of pericyte (PER) markers in clusters (**c**), as well as markers associated with matrix remodeling (**d**, top) and vascular functions (**d**, bottom) in pericyte subsets. **e**, Experimental scheme and UMAP projection of 12,171 LIN$^-$CD146$^+$ cardiac cells from *Cspg4*-CT2/tdTomato mice coloured by annotated subsets at day 7 PI. Violin plots depict normalized expression of *tdTomato* and *Cspg4*. LIN = CD45 and CD31. Ven. VSMC, venous/venular vascular smooth muscle cells; Art. VSMC, arterial/arteriolar vascular smooth muscle cells; PER, pericytes. **f-g**, Heatmaps illustrating relative average normalized expression of temporally regulated genes associated with inflammation in pericytes (**f**) and selected temporally regulated genes in VSMCs (**g**). Source data are provided as a Source Data file.

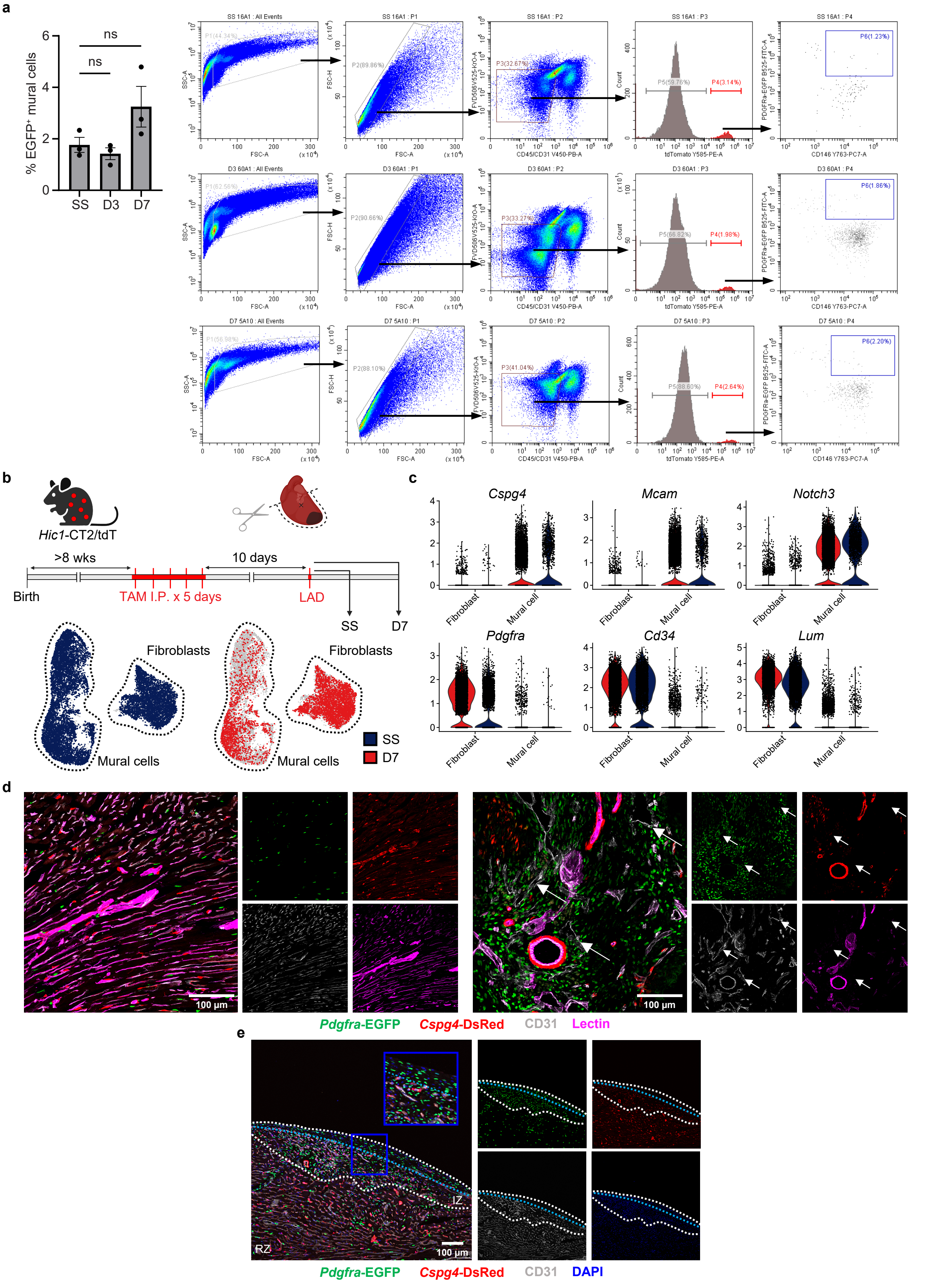
a
% EGFP⁺ mural cells
ns
ns
SS
D3
D7
SS 16A1 : All Events
SS 16A1 : P1
SS 16A1 : P2
SS 16A1 : P3
SS 16A1 : P4
D3 60A1 : All Events
D3 60A1 : P1
D3 60A1 : P2
D3 60A1 : P3
D3 60A1 : P4
D7 5A10 : All Events
D7 5A10 : P1
D7 5A10 : P2
D7 5A10 : P3
D7 5A10 : P4
P1(44.34%)
P2(89.86%)
P3(32.67%)
P5(59.76%)
P4(3.14%)
P6(1.23%)
P1(62.56%)
P2(90.66%)
P3(33.27%)
P5(66.82%)
P4(1.98%)
P6(1.86%)
P1(56.98%)
P2(88.10%)
P3(41.04%)
P5(88.60%)
P4(2.64%)
P6(2.20%)
SSC-A
FSC-A
FSC-H
FVD506V525-KrO-A
CD45/CD31 V450-PB-A
Count
tdTomato Y585-PE-A
PDGFRa-EGFP B525-FITC-A
CD146 Y763-PC7-A
b
Hic1-CT2/tdT
>8 wks
10 days
Birth
TAM I.P. x 5 days
LAD
SS
D7
Fibroblasts
Mural cells
Fibroblasts
Mural cells
SS
D7
c
Cspg4
Mcam
Notch3
Pdgfra
Cd34
Lum
Fibroblast
Mural cell
d
100 µm
100 µm
Pdgfra-EGFP
Cspg4-DsRed
CD31
Lectin
e
IZ
RZ
100 µm
Pdgfra-EGFP
Cspg4-DsRed
CD31
DAPI

Supp Fig 4. Lineage relationship of stromal subsets and assessment of vascular function. a, Assessment of *Pdgfra*-EGFP$^+$ lineage-traced mural cells using *Cspg4*-CT2/tdTomato/*Pdgfra*-EGFP mice (n = 3 per group pooled from 3 experiments). A one-way ANOVA with Tukey's multiple comparisons *post-hoc* test was used to compare the means of % CD146$^+$EGFP$^+$ cells out of LIN$^-$tdTomato$^+$ cells. NS, non-significant results. Representative gating strategy of all biological replicates per timepoint. LIN = CD45 + CD31. **b**, Experimental scheme and UMAP projection of LIN$^-$tdTomato$^+$ (with enrichment by CD146) cells from *Hic1*-CT2/tdTomato mice at SS and day 7 PI. LIN = CD45 + CD31. **c**, Normalized expression of known mural cell (top) and fibroblast (bottom) markers split by stromal cell type and coloured by timepoint. **d**, *In vivo* assessment of vascular perfusion in the remote (left) and infarct (right) zones by fluorophore-conjugated lectin (magenta) injection on day 14 PI. Heart sections were co-stained with CD31 (grey) in the presence of endogenous reporters, *Pdgfra*-EGFP (green) and *Cspg4*-DsRed (red). Arrows indicate instances of non-perfused vessels. Scale bar = 100 µm. Representative image of two biological replicates. **e**, Confocal image illustrating a two-layer division based on the distribution of vessels (CD31, grey), mural cells (*Cspg4*-DsRed, red), and fibroblasts (*Pdgfra*-EGFP, green) in the infarct zone at day 7 PI. Cell nuclei were counterstained with DAPI (blue). White and blue dotted lines delineate the infarct zone and its partition, respectively. Scale bar = 100 µm. Representative image of three biological replicates. Source data are provided as a Source Data file.

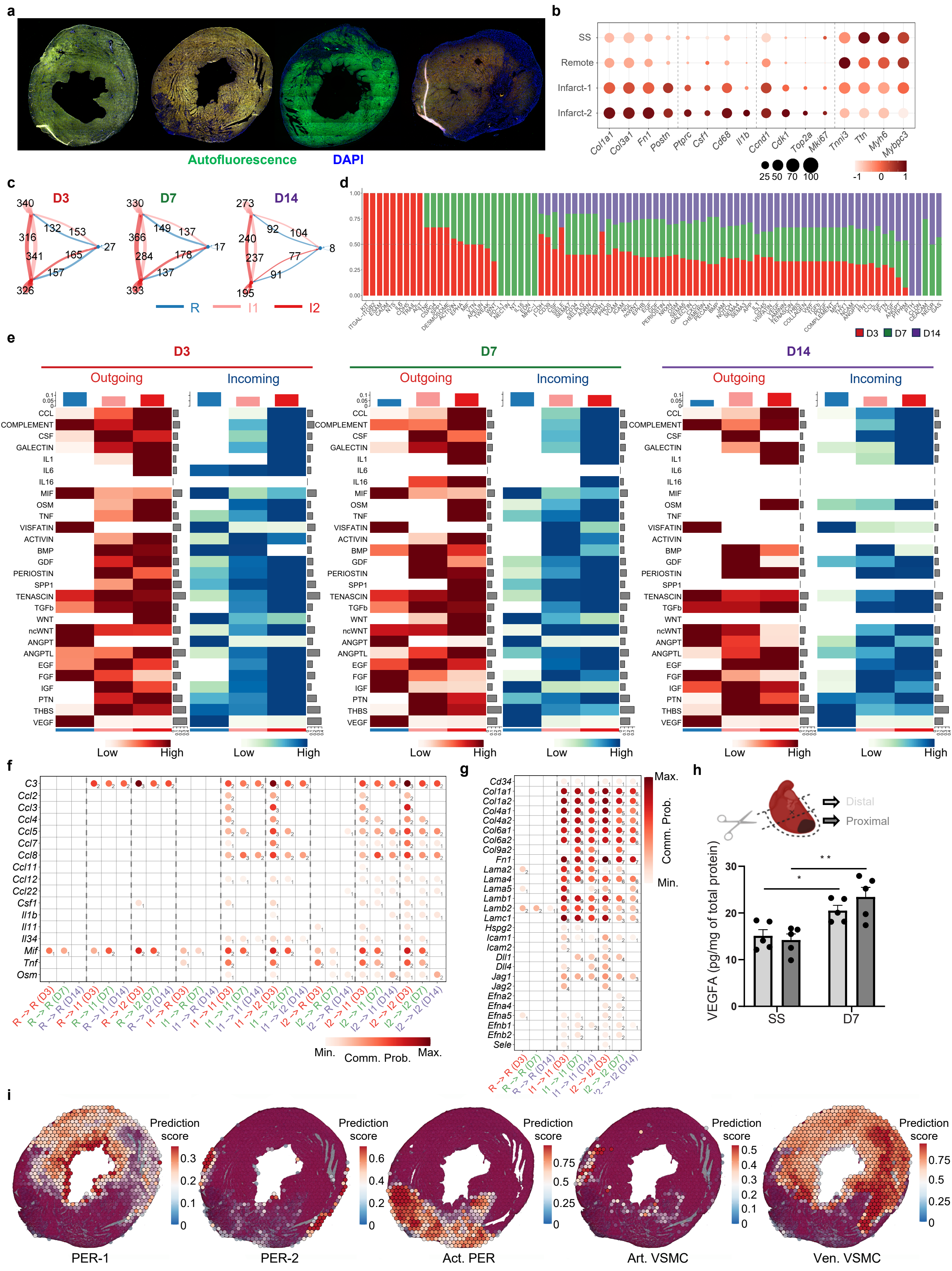

a
Autofluorescence
DAPI
b
SS
Remote
Infarct-1
Infarct-2
Col1a1
Col3a1
Fn1
Postn
Ptprc
Csf1
Cd68
Il1b
Ccnd1
Cdk1
Top2a
Mki67
Tnni3
Ttn
Myh6
Mybpc3
25
50
70
100
-1
0
1
c
D3
340
132
153
316
341
165
27
157
326
D7
330
149
137
366
284
178
17
137
333
D14
273
92
104
240
237
77
8
91
195
R
I1
I2
d
1.00
0.75
0.50
0.25
0.00
D3
D7
D14
e
D3
D7
D14
Outgoing
Incoming
CCL
COMPLEMENT
CSF
GALECTIN
IL1
IL6
IL16
MIF
OSM
TNF
VISFATIN
ACTIVIN
BMP
GDF
PERIOSTIN
SPP1
TENASCIN
TGFb
WNT
ncWNT
ANGPT
ANGPTL
EGF
FGF
IGF
PTN
THBS
VEGF
Low
High
f
C3
Ccl2
Ccl3
Ccl4
Ccl5
Ccl7
Ccl8
Ccl11
Ccl12
Ccl22
Csf1
Il1b
Il11
Il34
Mif
Tnf
Osm
R -> R (D3)
R -> R (D7)
R -> R (D14)
R -> I1 (D3)
R -> I1 (D7)
R -> I1 (D14)
R -> I2 (D3)
R -> I2 (D7)
R -> I2 (D14)
I1 -> R (D3)
I1 -> R (D7)
I1 -> R (D14)
I1 -> I1 (D3)
I1 -> I1 (D7)
I1 -> I1 (D14)
I1 -> I2 (D3)
I1 -> I2 (D7)
I1 -> I2 (D14)
I2 -> R (D3)
I2 -> R (D7)
I2 -> R (D14)
I2 -> I1 (D3)
I2 -> I1 (D7)
I2 -> I1 (D14)
I2 -> I2 (D3)
I2 -> I2 (D7)
I2 -> I2 (D14)
Min.
Comm. Prob.
Max.
g
Cd34
Col1a1
Col1a2
Col4a1
Col4a2
Col6a1
Col6a2
Col9a2
Fn1
Lama2
Lama4
Lama5
Lamb1
Lamb2
Lamc1
Hspg2
Icam1
Icam2
Dll1
Dll4
Jag1
Jag2
Efna2
Efna4
Efna5
Efnb1
Efnb2
Sele
R -> R (D3)
R -> R (D7)
R -> R (D14)
I1 -> I1 (D3)
I1 -> I1 (D7)
I1 -> I1 (D14)
I2 -> I2 (D3)
I2 -> I2 (D7)
I2 -> I2 (D14)
Max.
Comm. Prob.
Min.
h
Distal
Proximal
VEGFA (pg/mg of total protein)
30
20
10
0
**
*
SS
D7
i
Prediction score
0.3
0.2
0.1
0
PER-1
0.6
0.4
0.2
0
PER-2
0.75
0.5
0.25
0
Act. PER
0.5
0.4
0.3
0.2
0.1
0
Art. VSMC
0.75
0.5
0.25
0
Ven. VSMC

Supp Fig 5. Signaling networks underlying the infarcted heart. **a**, Confocal images of the heart sections used for positional transcriptomics after RNA extraction. DAPI (blue) was used as nuclear counterstain and tissue autofluorescence (green) represents regions of viable myocardium. **b**, Normalized gene expression of selected markers. Dot size and colour intensity represent the proportion of spots expressing the gene and the average normalized expression in each annotated region, respectively. **c**, Network diagrams depicting the number of active interactions amongst regions for each timepoint. Width of edges reflects relative number of interactions. R, remote zone; I1, Infarct-1; I2, Infarct-2. **d**, Relative information flow scaled to the number of active signaling pathways across timepoints. **e**, Heatmaps depicting the relative outgoing and incoming strengths of active signaling pathways. Top bars summarize the collective signaling strengths of pathways per region and side bars summarize the total signaling strengths of each pathway irrespective of regions. **f-g**, Directionality and signaling strength of ligands associated with inflammation (**f**) and selected non-secreted interactions (**g**). Dot colour intensity reflects the sum of communication probabilities of each ligand and the number of unique receptors associated with the ligand is supplemented. **h**, Graphical illustration of tissue collection and normalized VEGFA protein level in segments proximal and distal to the infarct. A two-way ANOVA with Šídák's multiple comparisons *post-hoc* test was used to compare the means of VEGFA level (n = 5). Data points derived from portions of the same sample are paired. P-value: 0.0427 and 0.001. Asterisks indicate statistically significant changes: $P < 0.05$ (*), $P < 0.01$ (**), $P < 0.001$ (***), $P < 0.0001$ (****). Data are presented as mean ± standard error of the mean. **i**, Prediction scores of annotated cell populations from scRNAseq data in the infarcted heart on day 3 PI. Ven. VSMC, venous/venular vascular smooth muscle cells; Art. VSMC, arterial/arteriolar vascular smooth muscle cells; PER, pericytes. Source data are provided as a Source Data file.
