# Supplementary material for: Spatiotemporal signaling underlies progressive vascular rarefaction in myocardial infarction
Source: Nat Commun. 2023 Dec 21;14:8498. doi: 10.1038/s41467-023-44227-6 (PMC10739910; doi:10.1038/s41467-023-44227-6)
Supplement: Supplementary file 2 — Description of Additional Supplementary Files [file 41467_2023_44227_MOESM2_ESM.pdf]

Description of Additional Supplementary Files

Supplementary Data 1. List of antibodies used for flow cytometry and immunofluorescence microscopy
