# Supplementary material for: Spatiotemporal signaling underlies progressive vascular rarefaction in myocardial infarction
Source: Nat Commun. 2023 Dec 21;14:8498. doi: 10.1038/s41467-023-44227-6 (PMC10739910; doi:10.1038/s41467-023-44227-6)
Supplement: Supplementary file 4 — Reporting Summary [file 41467_2023_44227_MOESM4_ESM.pdf]

nature portfolio

Corresponding author(s): Fabio Rossi

Last updated by author(s): Nov 28, 2023

Reporting Summary

Nature Portfolio wishes to improve the reproducibility of the work that we publish. This form provides structure for consistency and transparency in reporting. For further information on Nature Portfolio policies, see our Editorial Policies and the Editorial Policy Checklist.

Statistics

For all statistical analyses, confirm that the following items are present in the figure legend, table legend, main text, or Methods section.

n/a	Confirmed	
[ ]	[x]	The exact sample size (*n*) for each experimental group/condition, given as a discrete number and unit of measurement
[ ]	[x]	A statement on whether measurements were taken from distinct samples or whether the same sample was measured repeatedly
[ ]	[x]	The statistical test(s) used AND whether they are one- or two-sided<br>*Only common tests should be described solely by name; describe more complex techniques in the Methods section.*
[ ]	[x]	A description of all covariates tested
[ ]	[x]	A description of any assumptions or corrections, such as tests of normality and adjustment for multiple comparisons
[ ]	[x]	A full description of the statistical parameters including central tendency (e.g. means) or other basic estimates (e.g. regression coefficient) AND variation (e.g. standard deviation) or associated estimates of uncertainty (e.g. confidence intervals)
[ ]	[x]	For null hypothesis testing, the test statistic (e.g. *F*, *t*, *r*) with confidence intervals, effect sizes, degrees of freedom and *P* value noted<br>*Give P values as exact values whenever suitable.*
[x]	[ ]	For Bayesian analysis, information on the choice of priors and Markov chain Monte Carlo settings
[x]	[ ]	For hierarchical and complex designs, identification of the appropriate level for tests and full reporting of outcomes
[ ]	[x]	Estimates of effect sizes (e.g. Cohen's *d*, Pearson's *r*), indicating how they were calculated

Our web collection on statistics for biologists contains articles on many of the points above.

Software and code

Policy information about availability of computer code

Data collection	Zeiss Zen v.3.4 Blue Edition, NIS Elements (v.5.11.03), CytExpert (v.2.4), FlowJo (v.10.7.1)
Data analysis	Image processing and thresholding were performed in Fiji Image J (v.1.53q).<br>GraphPad Prism 9 was used to perform statistical analysis of quantified data from flow cytometry and image analysis.<br>Compilation of figures was performed in Adobe Illustrator 2021.<br>Processing of reads for scRNAseq and spatial transcriptomic data was performed using Cellranger (v.4.0.0) and SpaceRanger (v.2.0.1). Downstream analysis with Seurat (v.3.2.0), DAVID Bioinformatics Resources (2023), SCENIC (v.1.1.2-2), and CellChat (v.1.6.1) was performed with R (v.3.6.1 and 4.1.2).<br>All data processing and analysis scripts are constructed as per the vignettes of their respective sources and accessible on the GitHub repository [https://github.com/rossilab/spatial_temporal_atlas]. Please refer to methods above for details of key parameters and package versions.

For manuscripts utilizing custom algorithms or software that are central to the research but not yet described in published literature, software must be made available to editors and reviewers. We strongly encourage code deposition in a community repository (e.g. GitHub). See the Nature Portfolio guidelines for submitting code & software for further information.

Data

Policy information about availability of data

All manuscripts must include a data availability statement. This statement should provide the following information, where applicable:

- Accession codes, unique identifiers, or web links for publicly available datasets
- A description of any restrictions on data availability
- For clinical datasets or third party data, please ensure that the statement adheres to our policy

The single-cell and spatial transcriptomic data generated in this study have been deposited in the Gene Expression Omnibus (GEO) database under the accession code GSE206787 [https://www.ncbi.nlm.nih.gov/geo/query/acc.cgi?acc=GSE206787]. All databases associated with the softwares and packages used in the study are described in the Methods section. To promote readership, the processed data can be accessed and probed on our publicly accessible platform, Integrated Single-cell NAvigation Portal (ISNAP) [https://isnap.rossilab.dev/GSE206787/]. Source data are provided with this paper.

Research involving human participants, their data, or biological material

Policy information about studies with human participants or human data. See also policy information about sex, gender (identity/presentation), and sexual orientation and race, ethnicity and racism.

Reporting on sex and gender	N/A
Reporting on race, ethnicity, or other socially relevant groupings	N/A
Population characteristics	N/A
Recruitment	N/A
Ethics oversight	N/A

Note that full information on the approval of the study protocol must also be provided in the manuscript.

Field-specific reporting

Please select the one below that is the best fit for your research. If you are not sure, read the appropriate sections before making your selection.

[x] Life sciences   [ ] Behavioural & social sciences   [ ] Ecological, evolutionary & environmental sciences

For a reference copy of the document with all sections, see nature.com/documents/nr-reporting-summary-flat.pdf

Life sciences study design

All studies must disclose on these points even when the disclosure is negative.

Sample size	Sample size calculation was not performed in this study. Our lab's expertise in similar experiments served as a guide for selecting sample size needed to draw rational conclusions. This accounts for the nature of each type of experiment, which differs in the degree of variability and error generated for measurements. All scRNAseq experiments involved digestion of hearts and pooling (if needed) of cells to obtain sufficient number of cells for library preparation. For all flow cytometry experiments involving EdU incorporation, lineage tracing, and quantification of relative cell proportions, a sample size of at least three mice per group was used. For immunostaining experiments, we prepared one (with additional supporting evidence) to three biological replicates for claims requiring representative images and at least three biological replicates for claims requiring statistical tests. For ELISA, five biological replicates were used.
Data exclusions	Data exclusions were made for scRNAseq experiments based on: 1) doublet detection through cell hashing (using 0.99 quantile as a threshold for positive hashing, if applicable); 2) quality control metrics for each cell. A cell is removed if it expresses less than 200 genes, retains extreme number of UMIs compare to other cells in the dataset, and more than 10-15% of its transcripts are mitochondrial transcripts. No other data were excluded from analysis.
Replication	At least three animals were used for all experiments requiring quantification and statistical analysis. Descriptions on the number of biological replicates, the number of independent experiments, and error bars are included in the respective figure legends.
Randomization	Age-matched animals of both sexes were randomly assigned to different experimental groups wherever possible. This included all experiments requiring quantification and statistical analysis.
Blinding	Blinding was not performed in this study due to technical constraints of left anterior descending coronary artery ligation (LAD ligation) surgeries. While animals were randomly assigned a time point after surgery, the analyst of data was aware of the time point to which the animals were harvested.

Reporting for specific materials, systems and methods

We require information from authors about some types of materials, experimental systems and methods used in many studies. Here, indicate whether each material, system or method listed is relevant to your study. If you are not sure if a list item applies to your research, read the appropriate section before selecting a response.

Materials & experimental systems

n/a	Involved in the study
☐	☒ Antibodies
☒	☐ Eukaryotic cell lines
☒	☐ Palaeontology and archaeology
☐	☒ Animals and other organisms
☒	☐ Clinical data
☒	☐ Dual use research of concern
☒	☐ Plants

Methods

n/a	Involved in the study
☒	☐ ChIP-seq
☐	☒ Flow cytometry
☒	☐ MRI-based neuroimaging

Antibodies

Antibodies used

Flow cytometry/FACS sorting:
Anti-CD45 (fluorophore: eFluor-450, company: eBioscience, cat #: 48-0451-82, clone: 30-F11, lot #: E10032-1631)
Anti-CD45 (fluorophore: APC, company: eBioscience, cat #: 17-0451-83, clone: 30-F11, lot #: 2049099)
Anti-CD45 (fluorophore: FITC, company: eBioscience, cat #: 11-0451-85, clone: 30-F11, lot #: 4311552)
Anti-CD31 (fluorophore: eFluor-450, company: eBioscience, cat #: 48-0311-82, clone: 390, lot #: 4292978)
Anti-CD31 (fluorophore: APC, company: BD, cat #: 551262, clone: MEC 13.3, lot #: N/A)
Anti-CD31 (fluorophore: FITC, company: eBioscience, cat #: 11-0311-85, clone: 390, lot #: E00254-1633)
Anti-CD146 (fluorophore: PECy7, company: BioLegend, cat #: 134714, clone: ME-9F1, lot #: B272492)
Anti-CD146 (fluorophore: BV786 company: BD, cat #: 741037, clone: ME-9F1, lot #: 3079438)
Anti-CD39 (fluorophore: Alexa 647, company: BioLegend, cat #: 143808, clone: Duha59, lot #: B252739)
Anti-CD39 (fluorophore: PECy7,, company: BioLegend, cat #: 143805 clone: Duha59, lot #: B355060)

Immunostaining:
Anti-CD31 (unconjugated, company: R&D, cat #: AF3628, clone: polyclonal, lot #: YZU011G21)
Anti-Laminin (unconjugated, company: Abcam, cat #: AB11575, clone: polyclonal, lot #: GR3349807-1)
Anti-CNN1 (unconjugated, company: Abcam, cat #: AB46794, clone: EP798Y, lot #: GR33098521-1)
Anti-GFP (unconjugated, company: Abcam, cat #: AB5450, clone: polyclonal, lot #: GR306486-1)
Anti-ASMA (unconjugated, company: eBioscience, cat #: 14976082, clone: 1A4, lot #: 4280517)
Anti-GAL3 (unconjugated, company: BioLegend, cat #: 125401, clone: M3/38, lot #: WI327967)
Anti-NG2 (unconjugated, company: Sigma-Aldrich, cat #: AB5320, clone: Polyclonal, lot #: 3920775)
Anti-CD39 (unconjugated, company: Invitrogen, cat #: 14-0391-82, clone: 24DMS1, lot #: E03690-1630)

Donkey anti-chicken IgY (H+L) (fluorophore: FITC, company: Invitrogen, cat #: SA1-72000, clone: polyclonal, lot #: XA3468102)
Donkey anti-goat IgG (H+L) (fluorophore: Alexa Fluor 405, company: Abcam, cat #: AB175664, clone: polyclonal, lot #: GR3238419-2)
Donkey anti-goat IgG (H+L) (fluorophore: Alexa Fluor 488, company: Invitrogen, cat #: A11055, clone: polyclonal, lot #: 1827671)
Donkey anti-goat IgG (H+L) (fluorophore: Alexa Fluor 647, company: Invitrogen, cat #: A21447, clone: polyclonal, lot #: 2045332)
Donkey anti-rabbit IgG (H+L) (fluorophore: Alexa Fluor 488, company: Invitrogen, cat #: A21206, clone: polyclonal, lot #: 819556)
Donkey anti-rabbit IgG (H+L) (fluorophore: Alexa Fluor 647, company: Invitrogen, cat #: A31573, clone: polyclonal, lot #: 1903516)
Donkey anti-mouse IgG (H+L) (fluorophore: Alexa Fluor 555, company: Invitrogen, cat #: A31570, clone: polyclonal, lot #: 737680)
Donkey anti-rat IgG (H+L) (fluorophore: Alexa Fluor 647, company: Abcam, cat #: AB150155, clone: polyclonal, lot #: GR3420807-1)
Goat anti-rat IgG (H+L) (fluorophore: Alexa Fluor 647, company: Invitrogen, cat #: A21247, clone: polyclonal, lot #: 46411)

Validation

All primary antibodies used for flow cytometry/FACS sorting were validated prior and during the experiments with single-color controls and FMOs. Likewise, all immunostaining experiments were performed with negative controls (i.e. secondary staining only).

Flow cytometry/FACS sorting:
Anti-CD45 (company: eBioscience, cat #: 48-0451-82, clone: 30-F11, lot #: E10032-1631): The antibody has been validated by the manufacturer with mouse bone marrow cells via flow cytometric analysis and reacts with all isoforms of CD45. Cited in 127 references.
Anti-CD45 (company: eBioscience, cat #: 17-0451-83, clone: 30-F11, lot #: 2049099): The antibody has been validated by the manufacturer with mouse bone marrow cells and splenocytes via flow cytometric analysis and reacts with all isoforms of CD45. Cited in 137 references.
Anti-CD45 (company: eBioscience, cat #: 11-0451-85, clone: 30-F11, lot #: 4311552): The antibody has been validated by the manufacturer with mouse bone marrow cells via flow cytometric analysis and reacts with all isoforms of CD45. Cited in 393 references.
Anti-CD31 (company: eBioscience, cat #: 48-0311-82, clone: 390, lot #: 4292978): The antibody has been validated by the

manufacturer with mouse bone marrow cells via flow cytometric analysis. It is mostly expressed in endothelial cells. However, low expression was detected in immune cells and platelets. Cited in 98 references.
Anti-CD31 (company: BD, cat #: 551262, clone: MEC 13.3, lot #: N/A): The antibody has been routinely validated by the manufacturer with mouse bone marrow cells via flow cytometric analysis. Cited in 13 references.
Anti-CD31 (company: eBioscience, cat #: 11-0311-85, clone: 390, lot #: E00254-1633): The antibody has been validated by the manufacturer with mouse thymocytes and splenocytes via flow cytometric analysis and immunohistochemical staining in frozen tissues. It is mostly expressed in endothelial cells. However, low expression was detected in immune cells and platelets. Cited in 168 references.
Anti-CD146 (fluorophore: PECy7, company: BioLegend, cat #: 134714, clone: ME-9F1, lot #: B272492): The antibody has been validated by the manufacturer with mouse endothelial cells via flow cytometric analysis. Cited in 10 publications.
Anti-CD146 (fluorophore: BV786 company: BD, cat #: 741037, clone: ME-9F1, lot #: 3079438): The antibody has been routinely validated by the manufacturer in mouse lymph nodes via flow cytometric analysis. Cited in 4 publications.
Anti-CD39 (fluorophore: Alexa 647, company: BioLegend, cat #: 143808, clone: Duha59, lot #: B252739): The antibody has been validated by the manufacturer with mouse splenocytes via flow cytometric analysis. Cited in 3 publications.
Anti-CD39 (fluorophore: PECy7, company: BioLegend, cat #: 143805 clone: Duha59, lot #: B355060): The antibody has been validated by the manufacturer with mouse splenocytes via flow cytometric analysis. Cited in 12 publications.

Immunostaining:
Anti-CD31 (company: R&D, cat #: AF3628, clone: polyclonal, lot #: YZU011G21): The antibody has been validated by the manufacturer with immunostaining in mouse embryo and rat heart via Western blots, ELISAs, and flow cytometric assay. Cited in 226 references.
Anti-Laminin (company: Abcam, cat #: AB11575, clone: polyclonal, lot #: GR3349807-1): The antibody has been validated by the manufacturer in formalin-fixed human tissues via dot blot immunoassay and immunohistochemistry. It does not react with fibronectin, vitronectin, collagen IV, or chondroitin sulfates. Cited in 567 references.
Anti-CNN1 (company: Abcam, cat #: AB46794, clone: EP798Y, lot #: GR33098521-1): The antibody has been validated by the manufacturer in mouse cardiac muscle tissue sections and thoracic aortic smooth muscle cells via Western blot, immunohistochemistry, and immunofluorescence staining. Cited in 234 references.
Anti-GFP (company: Abcam, cat #: AB5450, clone: polyclonal, lot #: GR306486-1): The antibody has been validated by the manufacturer in murine brain tissue with endogenous GFP expression via immunoprecipitation, immunohistochemistry, electron microscopy, Western blot, and immunofluorescence staining. Cited in 210 references.
Anti-ASMA (company: eBioscience, cat #: 14976082, clone: 1A4, lot #: 4280517): The antibody has been validated by the manufacturer in formaldehyde-fixed human cells and tissue sections via immunohistochemistry and immunocytochemistry. Many publications have also reported positive staining using this antibody across mouse tissue types. Cited in 52 references.
Anti-GAL3 (company: BioLegend, cat #: 125401, clone: M3/38, lot #: WI327967): The antibody has been validated by the manufacturer in HeLa cells and BALB/c mouse peritoneal macrophages via flow cytometric assay, immunocytochemistry, and Western blot. Cited in 39 publications.
Anti-NG2 (unconjugated, company: Sigma-Aldrich, cat #: AB5320, clone: Polyclonal, lot #: 3920775): The antibody has been routinely validated by the manufacturer in rat brain lysates via Western blot. Cited in numerous publications.
Anti-CD39 (unconjugated, company: Invitrogen, cat #: 14-0391-82, clone: 24DMS1, lot #: E03690-1630): The antibody has been validated by the manufacturer in mouse splenocytes via flow cytometric assay and immunoblot analysis. Cited in 16 references.
Donkey anti-chicken IgY (H+L) (company: Invitrogen, cat #: SA1-72000, clone: polyclonal, lot #: XA3468102): A few publications have reported positive staining using this secondary antibody across mouse tissues including the hypothalamus. Cited in 10 references.
Donkey anti-goat IgG (H+L) (company: Abcam, cat #: AB175664, clone: polyclonal, lot #: GR3238419-2): The secondary antibody has been validated by manufacturer in HeLa cells. Cited in 20 references.
Donkey anti-goat IgG (H+L) (company: Invitrogen, cat #: A11055, clone: polyclonal, lot #: 1827671): Many publications have reported positive staining using this secondary antibody across mouse tissues including embryonic heart and hippocampus. Cited in 2279 references.
Donkey anti-goat IgG (H+L) (company: Invitrogen, cat #: A21447, clone: polyclonal, lot #: 2045332): Many publications have reported positive staining using this secondary antibody across mouse tissues including lung and endocrine gland. Cited in 783 references.
Donkey anti-rabbit IgG (H+L) (company: Invitrogen, cat #: A21206, clone: polyclonal, lot #: 819556): Many publications have reported positive staining using this secondary antibody across mouse tissues including skeletal muscle. Cited in 6075 references.
Donkey anti-rabbit IgG (H+L) (company: Invitrogen, cat #: A31573, clone: polyclonal, lot #: 1903516): Many publications have reported positive staining using this secondary antibody across mouse tissues including sciatic nerve and liver. Cited in 1428 references.
Donkey anti-mouse IgG (H+L) (company: Invitrogen, cat #: A31570, clone: polyclonal, lot #: 737680): Many publications have reported positive staining using this secondary antibody across mouse tissues including carotid arteries and cerebellum. Cited in 930 references.
Donkey anti-rat IgG (H+L) (company: Abcam, cat #: AB150155, clone: polyclonal, lot #: GR3420807-1): The secondary antibody has been validated by manufacturer in HeLa cells. Cited in 51 references.
Goat anti-rat IgG (H+L) (company: Invitrogen, cat #: A21247, clone: polyclonal, lot #: 46411): Many publications have reported positive staining using this secondary antibody across mouse tissues including carotid arteries. Cited in 616 references.

Animals and other research organisms

Policy information about studies involving animals; ARRIVE guidelines recommended for reporting animal research, and Sex and Gender in Research

Laboratory animals	All animals used in the study are Mus Musculus maintained on a C57BL/6 background. Adult mice ranging from two to six months in age are used for all experiments where litters of similar age are used in the same experiment as much as possible. All mice were housed under standard conditions with 12:12 light-dark cycle, 21-23 °C, and 40-60% humidity level in a pathogen-free facility. Specifically, these include the following transgenic mice purchased from The Jackson Laboratory and mice bred from within the facility:<br>1. B6.129S4-Pdgfratm11(EGFP)Sor/J (referred to as Pdgfra-EGFP; JAX stock #007669)

2. Tg(Cspg4-DsRed.T1)1Akik/J (referred to as Cspg4-DsRed; JAX stock # 008241)
3. B6.Cg-Gt(ROSA)26Sortm14(CAG-tdTomato)Hze/J (referred to as "tdTomato", JAX # 007914)
4. B6.129X1-Gt(ROSA)26Sortm1(EYFP)Cos/J (referred to as "YFP", JAX #006148)
5. B6.Cg-Tg(Cspg4-cre/Esr1*)BAkik/J (referred to as "Cspg4-CT2", JAX stock #008538)
6. Hic1-CT2 (for details, please refer to Scott, R. W., Arostegui, M., Schweitzer, R., Rossi, F. M. V., & Underhill, T. M. (2019). Hic1 Defines Quiescent Mesenchymal Progenitor Subpopulations with Distinct Functions and Fates in Skeletal Muscle Regeneration. Cell stem cell, 25(6), 797–813.e9)
7. B6.129S-Pdgfratm1.1(cre/ERT2)Blh/J (referred to as "Pdgfra-CT2", JAX stock #032770)

8. Pdgfra-EGFP/Cspg4-DsRed (generated from cross between 1. and 2.)
9. Hic1-CT2/tdTomato (generated from cross between 3. and 6.)
10. Hic1-CT2/tdTomato/PDGFRα-EGFP (generated from cross amongst 1., 3., and 6.)
11. Hic1-CT2/YFP (generated from cross between 4. and 6.)
12. Hic1-CT2/YFP/Cspg4-DsRed (generated from cross amongst 2., 4., and 6.)
13. Pdgfra-CT2/YFP/Cspg4-DsRed (generated from cross amongst 2., 4., and 7.)
14. Cspg4-CT2/tdTomato (generated from cross between 3. and 5.)
15. Cspg4-CT2/tdTomato/Pdgfra-EGFP (generated from cross amongst 1., 3., and 5.)

Wild animals

The study did not involve wild animals.

Reporting on sex

Findings of this study were made from mice of both sexes. We acknowledge the extensive reports of sex-specific differences in tissue repair and functional recovery after myocardial infarction in both humans and mice. For all experiments, both male and female mice were used as many experiments required complex genotypes. However, mice from each sex were distributed in similar numbers across groups wherever possible.

Field-collected samples

The study did not involve field-collected samples.

Ethics oversight

Animal maintenance and experimental procedures were conducted in accordance with the Animal Care Committee's approval and regulations at the University of British Columbia.

Note that full information on the approval of the study protocol must also be provided in the manuscript.

Plants

Seed stocks

N/A

Novel plant genotypes

N/A

Authentication

N/A

Flow Cytometry

Plots

Confirm that:

- [x] The axis labels state the marker and fluorochrome used (e.g. CD4-FITC).
- [x] The axis scales are clearly visible. Include numbers along axes only for bottom left plot of group (a 'group' is an analysis of identical markers).
- [x] All plots are contour plots with outliers or pseudocolor plots.
- [x] A numerical value for number of cells or percentage (with statistics) is provided.

Methodology

Sample preparation

Mice were anesthetized with 0.5 mg/g tribromoethanol (Avertin) IP. Once anesthetized, a horizontal incision was made above the sternum through both the skin and musculoskeletal layer to expose the heart. Following transcardiac perfusion with 20 mL of PBS-EDTA (2mM), whole hearts were excised and their atria were trimmed off. Excised ventricles were cut into 2 mm pieces and digested in Collagenase type II solution (Millipore Sigma C6885; 500uL per heart at 2.5 U/mL) containing 5 mM CaCl2 for 30 minutes at 37°C. Digested lysate was quenched with cold PBS and centrifuged at 140 g to remove the supernatant. This process was repeated twice followed by a second round of digestion in a solution (500 uL per heart) containing Collagenase D (Millipore Sigma 11088882001; 1.5 U/ml), Dispase II (Millipore Sigma 04942078001; 2.4 U/ml) and 5 mM CaCl2 for 1 hour at 37°C. Digested lysate was triturated by pipetting, quenched with cold FACS buffer (PBS containing 2 mM EDTA and 2% FBS), and filtered through 40-µm strainer filters. Filtrate was centrifuged at 500 g and washed again with cold FACS buffer prior to incubation with primary antibodies for 30 minutes at 4°C.

Instrument	Flow cytometry and FACS were performed on Beckman Coulter CytoFLEX or LSR-II and BD Influx, respectively.
Software	Flow cytometry data were analyzed using the software CytExpert (Beckman Coulter) or FlowJo (BD Biosciences).
Cell population abundance	Cell purities are determined by characterizing cell types and their respective numbers during scRNAseq analysis.<br><br>scRNAseq of cardiac stromal cells at physiological state and day 7 post-infarction<br>Cell abundance: ~65% mural cells and ~35% fibroblasts<br>Sorting strategy: live CD45-/CD31-/tdTomato cells in Hic1-CT2/tdTomato mice (enrichment with mural cells using CD146)<br>Purity: No contamination of non-stromal cell types<br><br>scRNAseq timeseries of mural cells post-infarction:<br>Cell abundance: ~97% mural cells<br>Sorting strategy: live CD45-/CD31-/CD146+ cells in Hic1-CT2/YFP mice<br>Purity: Minimal contamination from Schwann cells and fibroblasts<br><br>scRNAseq of mural cells at day 7 post-infarction in Cspg4-CT2 mice:<br>Cell abundance: ~86% mural cells, ~10% fibroblasts, ~4% Schwann cells<br>Sorting strategy: live CD45-/CD31-/CD146+ cells in Cspg4-CT2/tdTomato mice<br>Purity: Some contamination from Schwann cells and fibroblasts
Gating strategy	In all flow cytometry or FACS experiments, FSC and SSC gates of the start cell population were made on areas where intact, singlet cells are typically located. Further gating was made on LIN- (i.e. CD45-/CD31-) live cells (based on PI, Hoechst, or fixable viability dye staining). For all sorting strategies of scRNAseq experiments, please refer to the response above.<br><br>Evaluation of relative proportions between PER-1 and PER-2 at steady state:<br>Mice: Hic1-CT2/YFP/Cspg4-DsRed<br>Gating strategy: DsRed+CD39-CD146+/YFP+/LIN- (PER-1), DsRed-CD39-CD146+/YFP+/LIN- (PER-2)<br><br>Evaluation of cell proliferation with EdU incorporation post-infarction (timeseries):<br>Mice: Hic1-CT2/YFP<br>Gating strategy: CD146+/YFP-/LIN- (Hic1- mural cells/Schwann cells), CD146+/YFP+/LIN- (Hic1+ mural cells), CD146+/YFP-/LIN+ (endothelial cells), and CD146-/YFP+/LIN- (fibroblasts)<br><br>Evaluation of cell proliferation with EdU incorporation post-infarction (day 3 post-infarction):<br>Mice: Hic1-CT2/YFP<br>Gating strategy: CD39-/CD146+/YFP+/LIN- (pericytes), CD39+/CD146+/YFP+/LIN- (Ven. VSMC), and CD39+/CD146+/YFP-/LIN- (Art. VSMC).<br><br>Evaluation of mural cell lineage post-infarction:<br>Mice: Cspg4-CT2/tdTomato/Pdgfra-EGFP<br>Gating strategy: LIN-/tdTomato-/CD146+/EGFP+ (EGFP+ mural cells)<br><br>To correct for fluorescence spillovers, single-color controls were used for compensation. Boundaries of positive and negative staining of cell populations were determined based on the use of appropriate FMOs.

☒ Tick this box to confirm that a figure exemplifying the gating strategy is provided in the Supplementary Information.
